# Machine learning prediction of malaria vaccine efficacy based on antibody profiles

**DOI:** 10.1371/journal.pcbi.1012131

**Published:** 2024-06-07

**Authors:** Jacqueline Wistuba-Hamprecht, Bernhard Reuter, Rolf Fendel, Stephen L. Hoffman, Joseph J. Campo, Philip L. Felgner, Peter G. Kremsner, Benjamin Mordmüller, Nico Pfeifer

**Affiliations:** 1 Department of Computer Science, University of Tübingen, Tübingen, Germany; 2 Institute for Biomedical Informatics, University of Tübingen, Tübingen, Germany; 3 Institute of Tropical Medicine, University of Tübingen, Tübingen, Germany; 4 German Center for Infection Research, partner site Tübingen, Tübingen, Germany; 5 Centre de Recherches Médicales de Lambaréné, Lambaréné, Gabon; 6 Sanaria Inc., Rockville, Maryland, United States of America; 7 Antigen Discovery Inc., Irvine, California, United States of America; 8 Department of Medicine, University of California Irvine, Irvine, California, United States of America; 9 Department of Medical Microbiology, Radboud University Medical Center, Nijmegen, The Netherlands; Columbia University Irving Medical Center, UNITED STATES

## Abstract

Immunization through repeated direct venous inoculation of *Plasmodium falciparum* (Pf) sporozoites (PfSPZ) under chloroquine chemoprophylaxis, using the PfSPZ Chemoprophylaxis Vaccine (PfSPZ-CVac), induces high-level protection against controlled human malaria infection (CHMI). Humoral and cellular immunity contribute to vaccine efficacy but only limited information about the implicated Pf-specific antigens is available. Here, we examined Pf-specific antibody profiles, measured by protein arrays representing the full Pf proteome, of 40 placebo- and PfSPZ-immunized malaria-naïve volunteers from an earlier published PfSPZ-CVac dose-escalation trial. For this purpose, we both utilized and adapted supervised machine learning methods to identify predictive antibody profiles at two different time points: after immunization and before CHMI. We developed an adapted multitask support vector machine (SVM) approach and compared it to standard methods, i.e. single-task SVM, regularized logistic regression and random forests. Our results show, that the multitask SVM approach improved the classification performance to discriminate the protection status based on the underlying antibody-profiles while combining time- and dose-dependent data in the prediction model. Additionally, we developed the new f*E*ature di*S*tance ex*P*lainabilit*Y* (ESPY) method to quantify the impact of single antigens on the non-linear multitask SVM model and make it more interpretable. In conclusion, our multitask SVM model outperforms the studied standard approaches in regard of classification performance. Moreover, with our new explanation method ESPY, we were able to interpret the impact of Pf-specific antigen antibody responses that predict sterile protective immunity against CHMI after immunization. The identified Pf-specific antigens may contribute to a better understanding of immunity against human malaria and may foster vaccine development.

## Author summary

Developing an effective malaria vaccine is challenging. Malaria is a life-threatening disease caused by the plasmodium parasite, which has a complex multi-stage life-cycle and expresses several thousand proteins in a highly coordinated manner. To date, our understanding of the immune mechanisms mediating protection against *Plasmodium falciparum* (Pf) is incomplete. Proteome microarrays have been used earlier by our clinical collaboration partners to identify Pf-specific antibody profiles of malaria-naïve volunteers during immunization with attenuated Pf sporozoites (PfSPZ). We reused this data to compare the ability of three supervised machine learning methods to identify predictive antibody profiles after immunization and before controlled human malaria infection (CHMI). We adapted a multitask support vector machine (SVM) approach to analyze time-dependent Pf-induced antibody profiles from several time points in a single prediction model. Our multitask SVM approach outperforms the studied standard approaches in classification performance. Additionally, we developed a new explanation method, named f*E*ature di*S*tance ex*P*lainabilit*Y* (ESPY), to interpret the impact of Pf-specific antigens. We applied ESPY on the multitask SVM model and identified diverse Pf-specific antigen sets after immunization and before CHMI. Furthermore, we showed that the identified Pf-induced antibody profiles vary among protected and non-protected individuals who had been exposed to different doses of PfSPZ.

## Introduction

Malaria is a major health problem: alone in 2022 it caused more than 249 million cases and approximately 608,000 deaths [1]. *Plasmodium falciparum* (Pf) is the causal agent of almost all malaria-related deaths. Children, pregnant women and malaria-naïve subjects are at high risk of developing severe malaria, whereas adult residents of highly endemic areas develop immunity that protects from severe disease [2–4]. In addition, proof-of-concept studies have shown that experimental inoculation of high doses of attenuated Pf sporozoites (PfSPZ) (the mosquito-to-human transmission stage of the parasite) can lead to sterile protection [5]. Nevertheless, developing an effective vaccine for Pf remains a huge challenge. Pf is genetically highly divers, employs several immune evasion strategies and has a complex, multi-stage life-cycle, during which more than 5,300 genes are expressed [6]. As a result, our understanding of immune responses to Pf-specific antigens that mediate naturally acquired or experimentally induced protection is incomplete.

It has been shown that up to 100% protection against controlled human malaria infection (CHMI) can be achieved by immunization of malaria-naïve adults by direct venous inoculation (DVI) of radiation-attenuated Pf sporozoites (Sanaria PfSPZ Vaccine) [7] and by chemo-attenuated PfSPZ (Sanaria PfSPZ-CVac) [8–10]. In those studies, protection is defined as an immune state that prevents parasites from reaching the blood stage, whereas in non-protected volunteers (either non-immunized or not successfully immunized participants) parasites will invade red blood cells following an approximately 6-day-long liver stage [8]. Only the asexual blood stage of the parasite is responsible for the symptoms and complications of malaria. Pf-specific protein microarrays can be used to characterize the pattern of antibody reactivity to Pf-specific epitopes. In [8] we used a Pf-specific protein microarray with 7,455 protein fragments, representing about 91% of the Pf proteome, to determine the antibody reactivity profile of 40 immunized and non-immunized malaria-naïve individuals after immunization and before CHMI. In this previous study [8], we showed, that among the subjects who received the highest dose of attenuated PfSPZ, all of whom were protected, twenty-two proteins were recognized on the Pf proteome microarray by more than half of the protected subjects [8]. A limitation of protein microarrays is that antibody reactivity profiles are characterized by a huge number of features in a comparatively small number of samples, a problem that is better known as the curse of dimensionality. Since machine learning methods became a famous choice for analyzing such high-dimensional data [11–13], we sought to complete our previous, primarily descriptive analyses of the data [8] to better understand and even predict PfSPZ-CVac-induced protective immunity.

Therefore, we adapted here a multitask support vector machine (multitask SVM) approach to identify predictive Pf-specific antibody profiles of protected and non-protected vaccinees and controls by integrating time- and dose-dependent data in a single prediction model. Combining related tasks into a single prediction model is more promising than training independent models with the data of each task, if the number of features is much greater than the number of samples [14]. SVM kernels can be used to model relationships between single related tasks and combine them into a sole prediction model—the multitask SVM.

Analysing such a large array of antibody profiles using a proteome microarray, strong correlations can be assumed, e.g., between fragments representing one protein, similar epitopes, and due to cross-reactivity [15]. In general, it is advised to remove strongly linearly correlated features, to avoid biasing the variable importance measure of the features [13] and to improve classification performance [16, 17]. Therefore, we assessed the classification performance of our adapted multitask SVM approach under conditions in which highly linearly correlated features were removed, and compared it to state-of-the-art methods, such as regularized logistic regression (RLR) [11], a standard SVM model with radial basis function (RBF) kernel, and a random forest (RF) approach. All these methods are known to be able to deal with high-dimensional data for the classification of protected versus non-protected vaccinees and controls [11, 13]. To enable an optimal comparison, we trained the three state-of-the-art methods, RLR, RF and RBF-SVM, respectively, either time-point-wise (task-wise) or time-point-combined (multi-time). In the former one, samples that belonged to different time points (that is, before and after immunization) were separately used to train the models, whereas in the latter one, the samples from both time points were combined to train the models (for more details see the materials and methods section). Our results show that the adapted multitask SVM approach improves the prediction performance when classifying protected PfSPZ-CVac vaccinees versus non-protected PfSPZ-CVac vaccinees and controls. Moreover, we can show that highly correlated features degraded classification performance of the state-of-the-art methods compared to our multitask SVM approach.

To identify and interpret informative features, i.e., single Pf-specific antigens, from the non-linear multitask SVM model, explainability models for non-linear machine leaning models are needed, which motivated us to develop the f*E*ature di*S*tance ex*P*lainabilit*Y* (ESPY) method. ESPY is inspired by a feature importance measure for sequence-based non-linear predictions [18]. The ESPY values are directly derived from a multitask SVM (or general SVM) model. ESPY uses systematically and specifically triggered changes in the distance of a consensus sample to the classification boundary (the boundary that separates the datapoints into two sets, one of each class in a binary classification scenario) of the SVM to estimate the importance of features (for more details see the materials and methods section), but could be extended to any machine learning model that provides classification scores indicating to how certain a classification is. Consequently, we identified individual informative Pf-specific antigens by their respective ESPY value for protected PfSPZ-CVac vaccinees and non-protected PfSPZ-CVac vaccinees and controls. Additionally, we compared our ESPY method with the SHapley Additive exPlanation (SHAP) framework from Lundberg et al. [19] on simulated data. The SHAP framework is an additive unified approach to derive feature importance values. The results of both explainability methods are similar on the simulated data, however ESPY significantly outperformed SHAP in run time: ESPY needed only a few seconds to compute feature importance values on the simulated data, while SHAP ran for more than 13 hours. Using our newly developed ESPY method, we show how to address the problem of explaining the predictions from a non-linear multitask SVM model based on single features. In summary, our adapted multitask SVM approach represents a classification method to integrate time- and dose-dependent data into a single prediction model, while ESPY provides explainability by means of identifying and evaluating informative single features from a non-linear model.

## Results

This section is structured into three main parts. In the first part, we show that our new multitask SVM approach can be used to classify with high accuracy protected PfSPZ-CVac vaccinees versus non-protected PfSPZ-CVac vaccinees and controls based on a subset of antibody (ab) intensity signals and excluding those that strongly linearly correlated above a Pearson correlation coefficient of *pcc* = 0.8 for both time points (post-immunization and pre-CHMI). We highlight the classification performance of our multitask SVM approach at each single time point for both the whole Pf-specific proteome microarray and a selection of cell-surface Pf-antigens compared to standard machine learning approaches. To enable this comparison, we trained the standard machine learning models either on each single time point separately or on a combined set of all ab signal intensities for both time points and PfSPZ doses together.

In the second part, we illustrate how the ESPY values are used to quantify which Pf-specific antigens are informative in classifying protected PfSPZ-CVac vaccinees versus non-protected PfSPZ-CVac vaccinees and controls.

The third and last part of this section shows ESPY values for simulated data and compares those with the SHAP values from Lundberg et al. [19].

In the following lines, for a better understanding of our results, we briefly summarize which data we used and how we applied our methods (for more details please refer to the materials and methods section).

To compare our new multitask SVM approach with standard machine learning methods, i.e., standard single-task SVM, RLR, and RF, we used the Pf-specific antibody reactivity profile from the earlier published PfSPZ-CVac clinical trail by [8]. The Pf-specific ab reactivity profile contains Pf-specific ab-mediated responses of 40 individuals at two different time points (post-immunization and pre-CHMI). The 40 individuals were vaccinated with different doses of PfSPZ-CVac: placebo (n = 13), 3.2 × 10^3^ PfSPZ (n = 9), 1.28 × 10^4^ PfSPZ (n = 9), 5.12 × 10^4^ PfSPZ (n = 9). For each individual the protection status, i.e., protected or non-protected, was assessed by CHMI. The comparison of the classification performance and the identification of the informative Pf-specific antigens was done at each single time point (post-immunization and pre-CHMI) through the overall study. Antibody responses after CHMI (post-CHMI) were excluded from our malaria vaccine efficacy prediction analysis since controls underwent a CHMI at this time point as well, making it unfeasible to apply binary classification, due to the lack of non-protected controls.

### Classification of protected PfSPZ-CVac vaccinees versus non-protected PfSPZ-CVac vaccinees and controls from the PfSPZ-CVac mediated antibody response

We used a multitask SVM approach to build our prediction model. The multitask SVM builds upon a multitask kernel matrix that is constructed from the single-task kernels matrices via element-wise multiplication (see materials and methods section). A critical step in building a multitask SVM model is to find the right combination of single-task kernels to encode the relationships found in the input data. We explored the following combinations of a time point kernel, an antibody signal kernel, and a dose kernel: the time point kernel matrix was calculated using a RBF kernel function, while the antibody signal and the dose kernels were either calculated using a polynomial or RBF kernel function. The prediction performance, the PR-AUC score (*Area Under the Precision Recall Curve*) of each studied kernel combination at each single time point was assessed via a 10-times repeated nested stratified 5-fold cross-validation over a grid of kernel parameters Table A in S1 Appendix. Due to the fact, that in our datasets the number of features outweighs the number of samples, we removed strongly linear correlated features above a Pearson correlation coefficient of *pcc* = 0.8 for the whole and a selection of cell-surface antibody reactivity profile, as earlier described by [13]. For details please refer to the materials and methods section.

**Fig 1:**
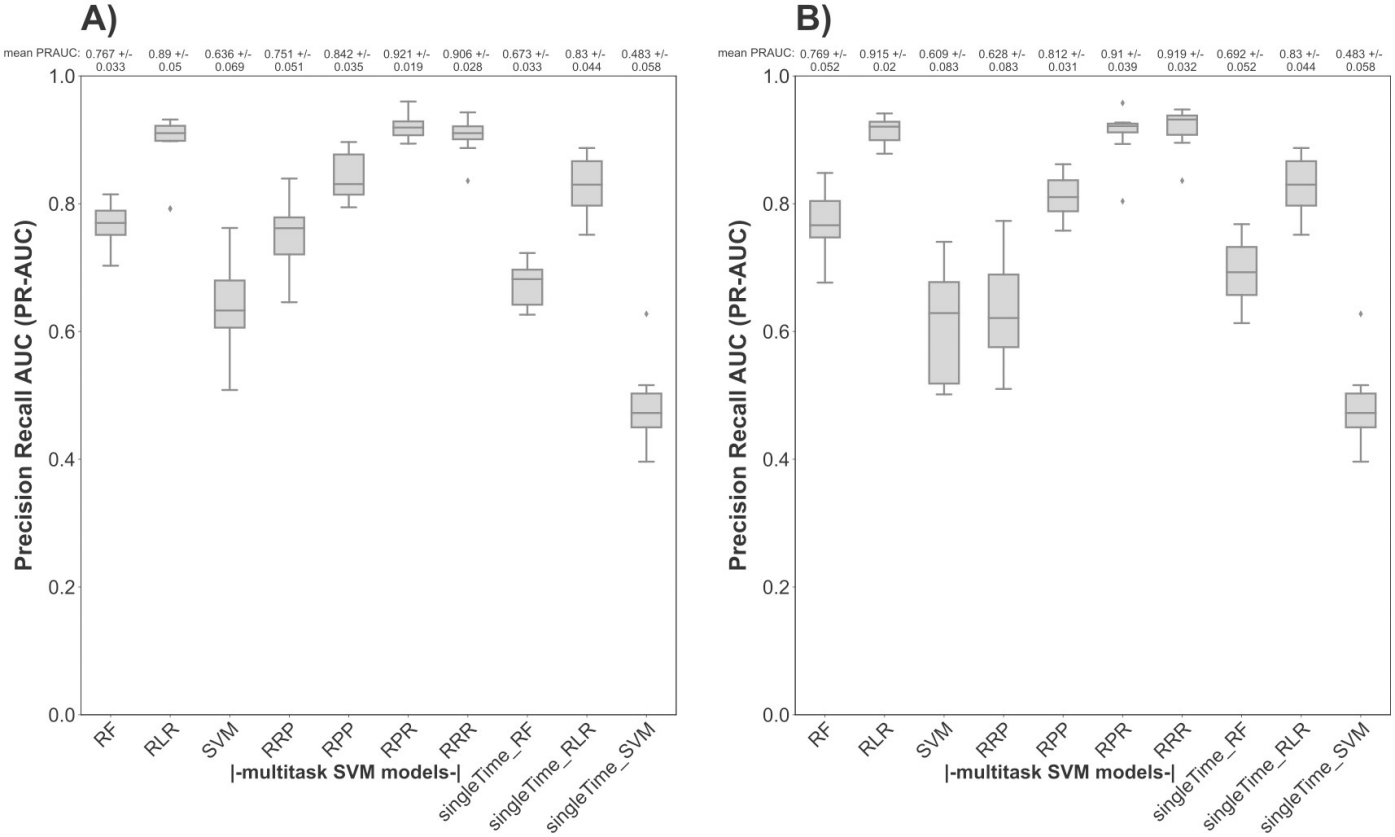
Performance of multitask SVM models in predicting the protection status based on the antibody reactivity profile per time point as compared to state-of-the-art approaches. The PR-AUC score of the RF, the RLR, the single-task SVM (trained either on each single time point or on the combined time points), and the multitask SVM model (using different combinations of kernel functions) for predicting the protection status based on the whole proteome antibody profile per time point was assessed via 10-times repeated nested stratified 5-fold cross-validation. RF, RLR and single-task SVM models trained on each time point separately are labeled by the extension ‘singleTime’. The mean PR-AUC score together with the standard deviation is displayed above each boxplot, with PR-AUC = 1 equating to perfect prediction and PR-AUC = 0.5 equating to random guessing. The PR-AUC performance of the different applied models is shown (A): at post-immunization and (B): at pre-CHMI.

Based on prediction performance comparisons between the studied kernel combinations (Table A in S1 Appendix), the combination of three RBF kernels (RRR) and the combination of RBF and polynomial kernels (RPR) into a multitask kernel matrix resulted in the highest nested cross-validation PR-AUC score compared to all other kernel combinations (Fig 1). First of all, we compared our multitask SVM approach with three state-of-the-art approaches, namely RLR (with elastic net regularization), RF and single-task RBF-SVM at each single time point based on the complete antibody reactivity profile (Fig 1). Moreover, to illustrate the differences in classification performance between our multitask SVM approach and state-of-the-art approaches we trained the state-of-the-art approaches either on each time point separately (stated by the extension “singleTime” for simplicity) (n = 40) or on the combined time points (n = 80). For details please refer to the materials and methods section. As shown in (Fig 1), the highest mean PR-AUC scores of the multitask SVM approaches were achieved by the kernel combinations RRR and RPR after immunization (Fig 1A) and before CHMI (Fig 1B) compared to the single-task RBF-SVM, the RLR and RF model trained on each time point separately or on the combined time points. Only before CHMI (Fig 1B) the RLR model trained on both time points achieved a similar mean PR-AUC score as the multitask SVM model for the RPR kernel combinations. The prediction performance of the state-of-the-art models, RF, RLR and single-task RBF-SVM was also assessed via a 10-times repeated nested stratified 5-fold cross-validation per time point (as detailed in the materials and methods section).

Finally, for a comprehensive view we compared the classification performances of our multitask SVM model and the state-of-the-art approaches for different Pearson correlation coefficients. As shown in Fig A in S1 Appendix, our multitask SVM model (for the kernel combinations RPR and RRR) is robust in its PR-AUC scores with less variance over different Pearson correlation coefficients in comparison to the state-of-the-art approaches after immunization (Fig A A in S1 Appendix) and before CHMI (Fig A B in S1 Appendix).

**Fig 2:**
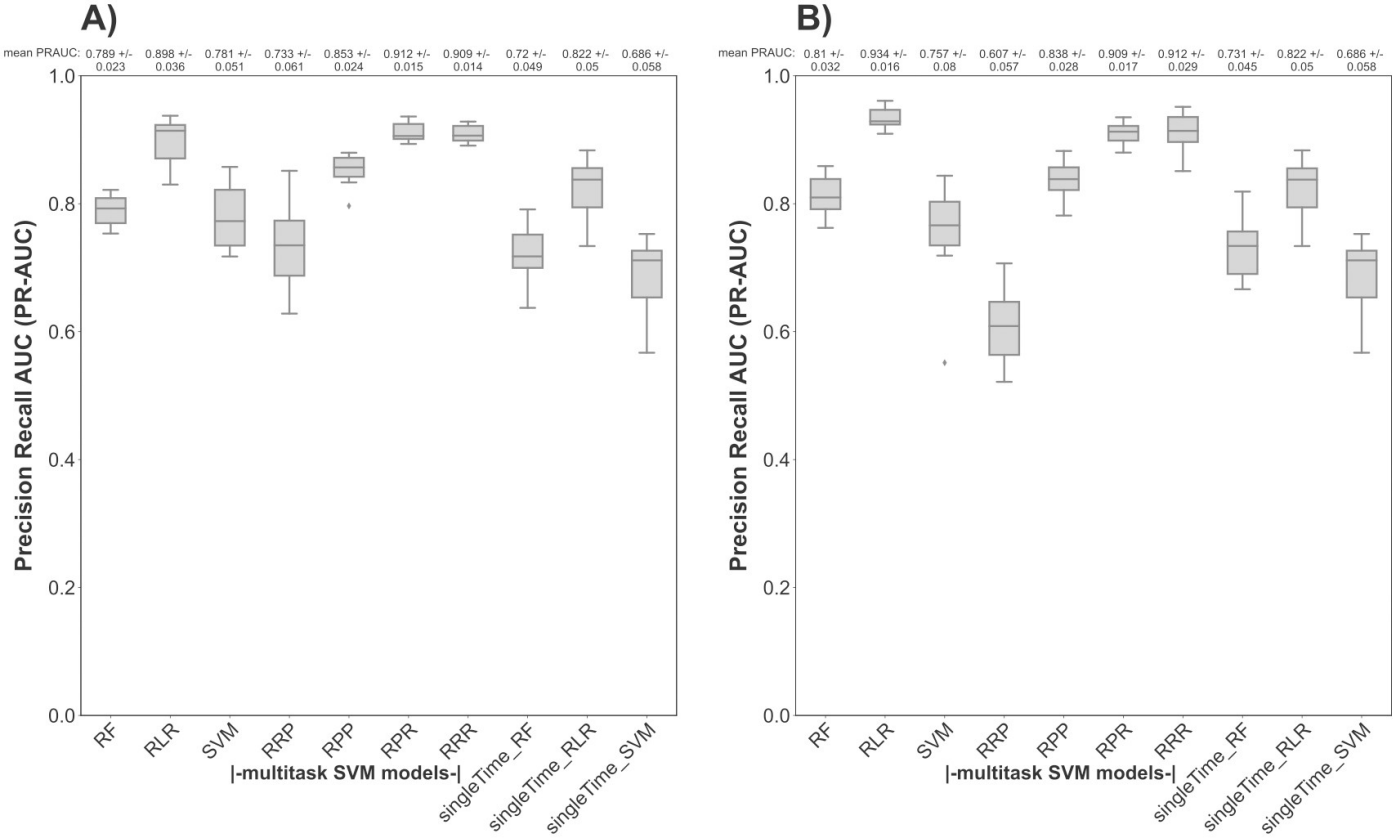
Performance of multitask SVM models in predicting the protection status based on cell-surface antibody reactivity profile per time point as compared to to state-of-the-art approaches. The PR-AUC score of the RF, the RLR, the single-task SVM (trained either on each single time point or on the combined time points), and the multitask SVM model (using different combinations of kernel functions) for predicting the protection status based on the selective cell-surface proteome antibody profile per time point was assessed via 10-times repeated nested stratified 5-fold cross-validation. RF, RLR and single-task SVM models trained on each time point separately are labeled by the extension ‘singleTime’. The mean PR-AUC score together with the standard deviation is displayed above each boxplot, with PR-AUC = 1 equating to perfect prediction and PR-AUC = 0.5 equating to random guessing. The PR-AUC performance of the different applied models is shown (A): at post-immunization, (B): at pre-CHMI.

In a second step, we used our multitask SVM approach to analyze the antibody profile against pre-selected Pf-specific cell-surface antigens. Fig 2 illustrates the prediction performance (PR-AUC) of the compared models based on the measured selective cell-surface antibody reactivity profile per time point. Again, the combination of three RBF kernels (RRR) and the combination of RBF and polynomial kernels (RPR) into a multitask kernel matrix resulted in the highest nested cross-validation PR-AUC score compared to all other kernel combinations at post-immunization (Fig 2A) and pre-CHMI (Fig 2B). The single-task RBF-SVM, trained either on each single time point separately or on the combined time points, performed relatively poorly in comparison to the multitask SVM approach. The RLR model trained on the combined time points achieved a higher mean PR-AUC score only at pre-CHMI (Fig 2B) compared to the multitask SVM approach. However, at post-immunization (Fig 2A) the RLR approach achieved lower mean PR-AUC values.

For the selective dataset, we could also show, that our multitask SVM approach (for the kernel combinations RPR and RRR) is robust in its PR-AUC scores with less variance over different Pearson correlation coefficients in comparison to the state-of-the-art approaches after immunization (Fig B A in S1 Appendix) and before CHMI (Fig B B in S1 Appendix).

Overall these results demonstrate that our multitask SVM approach provides state-of-the-art performance in classifying protected PfSPZ-CVac vaccinees versus non-protected PfSPZ-CVac vaccinees and controls per time point by combining different tasks into a single model. Also, strongly linear correlated features have only a small effect on the prediction performance of our multitask SVM approach in comparison to the state-of-the-art approaches (Figs A and B in S1 Appendix).

### Informative Pf-specific antigens for successful classification of protected PfSPZ-CVac vaccinees versus non-protected PfSPZ-CVac vaccinees and controls

Evaluation of informative Pf-specific antigens to exhibit an antibody profile at post-immunization and pre-CHMI is essential for predicting and improving vaccine-induced protective immunity. To identify antibody profiles from the underlying PfSPZ-CVac dataset, where the number of Pf-specific antigens (p = 7,455) is much higher than the number of individuals (n = 40), we combined data from several time points and tasks into a non-linear multitask model and identified and evaluated informative Pf-specific antigens per time point from this model (for details please refer to the materials and methods section). First, we evaluated the best parameter setting for the kernel combinations in the multitask SVM models by a 10-times repeated stratified 5-fold grid-search cross-validation for both the whole proteome microarray and the pre-selected set of cell-surface antigens. The best kernel combinations and the associated parameters were selected based on the highest mean PR-AUC score per time point. Again the kernel combinations of the “RPR” and “RRR” performed equally well (achieving the highest mean PR-AUC scores), whereas the kernel combination “RRR” resulted in a more stable prediction performance with lower standard deviation than the kernel combination “RPR” for the multitask SVM approach. Table B in S1 Appendix shows the combinations of kernels and the associated parameters of the multitask SVM that achieved the highest mean AUC score, both based on the whole Pf-specific proteome microarray and the pre-selected cell-surface antigens. The kernel combinations of choice for both datasets were the RBF kernel for time point similarity, the RBF kernel for the antibody signal intensity similarity, and the RBF kernel for the PfSPZ-specific dose similarity at post-immunization and pre-CHMI.

**Fig 3:**
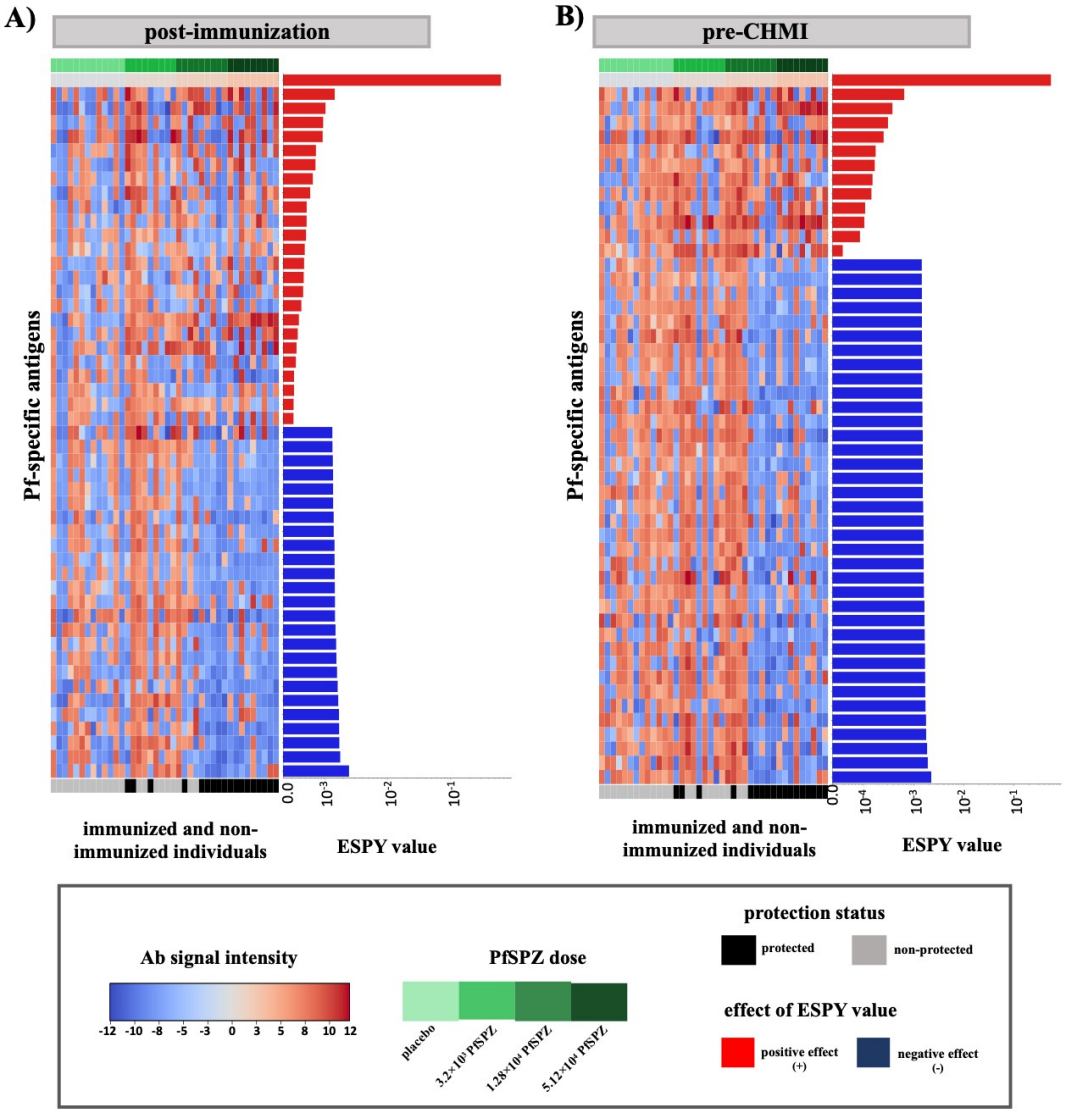
Antibody profile of protected and non-protected vaccinees and the placebo group against informative Pf-specific antigens. Informative Pf-specific antigens of the complete proteome microarray were evaluated at post-immunization and pre-CHMI. Pf-specific antigens identified to be important by ESPY evaluation showed either a high antibody signal intensity in protected vaccinees or unprotected vaccinees and controls. The top 50 Pf-specific antigens with the highest ESPY values are shown (A) at post-immunization and (B) at pre-CHMI. The heatmap plot shows the antibody signal intensity, while the bars on the right side of each figure show the importance and effect of each feature based on the ESPY value. ESPY values of Pf-specific antigens, that were evaluated to have a positive effect on the protection status classification are colored in red, while blue-colored bars represents antigens, that have a negative effect.

Second, we evaluated the Pf-induced antibody profiles of all individuals based on immunoreactivity to 574 Pf-specific antigens after removing strongly linearly correlated features above a Pearson correlation coefficient of *pcc* = 0.8. Fig 3 shows the antibody profile (at post-immunization and pre-CHMI) for the top 50 informative Pf-specific antigens selected using the ESPY values from the multitask SVM model. To better understand the informativeness of evaluated Pf-specific antigens in the classification of protected versus non-protected vaccinees and controls, the ESPY value has two properties: the absolute ESPY value of a Pf-specific antigens reflects how much a single Pf-specific antigen affected the prediction. Whereas, the *effect* label reflects the direction on the classification. A positive *effect* “ + ” denotes that the evaluated informative feature is more similar to the positive sample, and vice versa for a negative *effect* “ − ”.

Immunized protected individuals, who received the highest dose of PfSPZ-CVac, showed a higher antibody reactivity against CSP, PfEMP1, MSP2, MSP4, LSA1, conserved (membrane) proteins with unknown function, and intra-cellular/trans-membrane proteins, at post-immunization. The identified Pf-specific antigens were assessed to have a positive effect (red bars) on the classification of protected versus non-protected individuals (Fig 3A) by ESPY evaluation and are therefore more similar to protected vaccinees. The informative Pf-specific antigens with the highest ESPY value were membrane proteins, conserved proteins of unknown function and intracellular proteins. This evaluation was done considering, the PfSPZ-dose as an informative feature with the highest ESPY value and a positive effect on the classification of protected vaccinees versus non-protected vaccinees and controls at both time points and datasets.

Zinc finger protein, PHISTb, PfEMP1, intracellular proteins, and proteins of unknown function were evaluated (by ESPY) to have a negative effect (blue bars) in the protection status classification after immunization (Fig 3A) in immunized individuals who received a lower dose or were located in the placebo group.

At pre-CHMI (Fig 3B), a smaller set of twelve Pf-specific antigens, namely CSP, LSA1, GLURP and proteins of unknown function were evaluated to have a positive effect on the classification of protected vaccinees versus non-protected vaccinees and controls, and showed a higher antibody signal intensity in immunized protected individuals. By contrast, all other informative Pf-specific antigens, like zinc finger protein, ETRAMP5, PfEMP1, and mostly conserved proteins of unknown function and intracellular proteins were evaluated to have a negative effect on the protection status classification, and showed a higher antibody signal intensity in non-protected vaccinees and controls. The top 50 informative Pf-specific antigens for the whole proteome microarray at post-immunization and pre-CHMI based on ESPY evaluation, are listed in Tables C and D in S1 Appendix.

**Fig 4:**
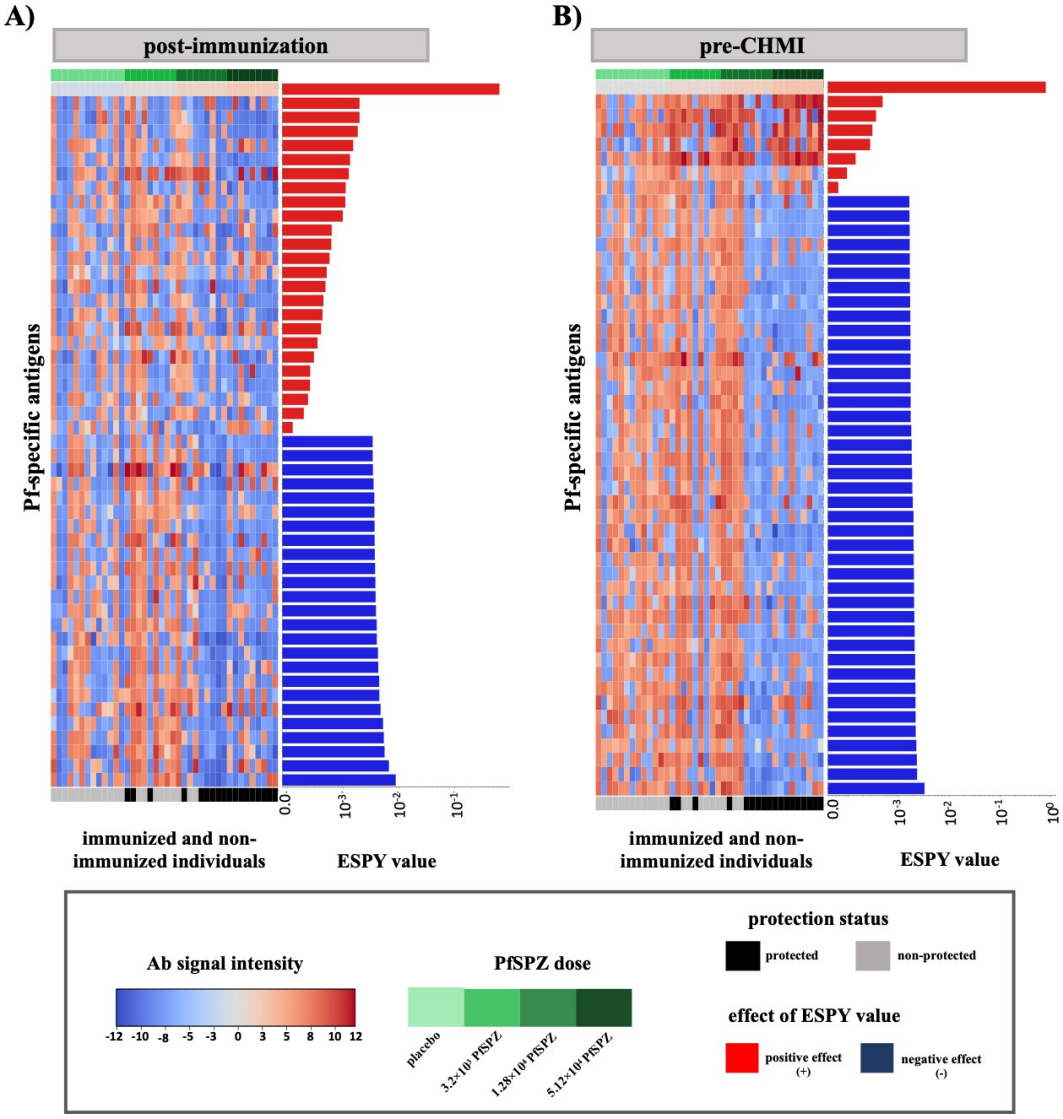
Antibody profile of protected and non-protected vaccinees and the control group against informative cell-surface Pf-specific antigens. Informative Pf-specific antigens against pre-selected Pf-specific cell-surface antigens were evaluated at post-immunisation and pre-CHMI. Pf-specific antigens identified to be important by ESPY evaluation showed either a high antibody signal intensity in protected vaccinees or unprotected vaccinees and controls. The top 50 Pf-specific antigens with the highest ESPY values are shown (A) at post-immunization and (B) at pre-CHMI. The heatmap plot shows the antibody signal intensity, while the bars on the right side of each figure show the importance and effect of each feature based on the ESPY value. ESPY values of Pf-specific antigens that were evaluated to have a positive effect on the protection status classification are colored in red, while blue-colored bars represent antigens that have a negative effect.

Third, we evaluated Pf-induced antibody profiles of all individuals using immunoreactivity to the pre-selected 188 Pf-specific cell surface antigens after removing strongly linearly correlated features above a Pearson correlation coefficient of *pcc* = 0.8. Fig 4 shows the antibody profiles (at post-immunization and pre-CHMI) for the top 50 informative Pf-specific cell-surface antigens based on ESPY evaluation of the multitask SVM model. At post-immunization (Fig 4A) MSP2, PfEMP1, rifin and membrane proteins of unknown function were evaluated to have a positive effect on the classification of protected vaccinees versus non-protected vaccinees and controls. By contrast, mainly zinc finger protein, PHISTb, PHISTc, PfEMP1 and conserved proteins of unknown function were evaluated to have a negative effect on the protection status classification.

At pre-CHMI, again a small set of seven informative Pf-specific antigens, namely CSP, MSP2, PfEMP1, LSA1 and (membrane) proteins of unknown function were evaluated to have a positive effect on the classification of protected vaccinees versus non-protected vaccinees and controls. All other Pf-specific antigens were evaluated to have a negative effect on the protection status classification. Those were mainly rifin, ETRAMP5, PHISTb, PHISTc, zinc finger proteins, (membrane) proteins of unknown function, PfEMP1 and MSP7, and showed a higher antibody signal intensity in unprotected vaccinees and the control group (Fig 4B).

Overall, the identified informative Pf-specific cell-surface antigens are well-known pre-erythrocytic and erythrocytic Pf-specific antigens, like CSP, ETRAMP, MSP, LSA, PfEMP1, PHISTb/c, rifin, zinc finger protein, and other Pf-specific antigens of unknown function. The top 50 informative Pf-specific antigens for the pre-selected cell-surface proteome microarray at post-immunization and pre-CHMI based on the ESPY value are listed in Tables E and F in S1 Appendix.

### ESPY versus SHAP: Evaluation of informative features on simulated data

**Fig 5:**
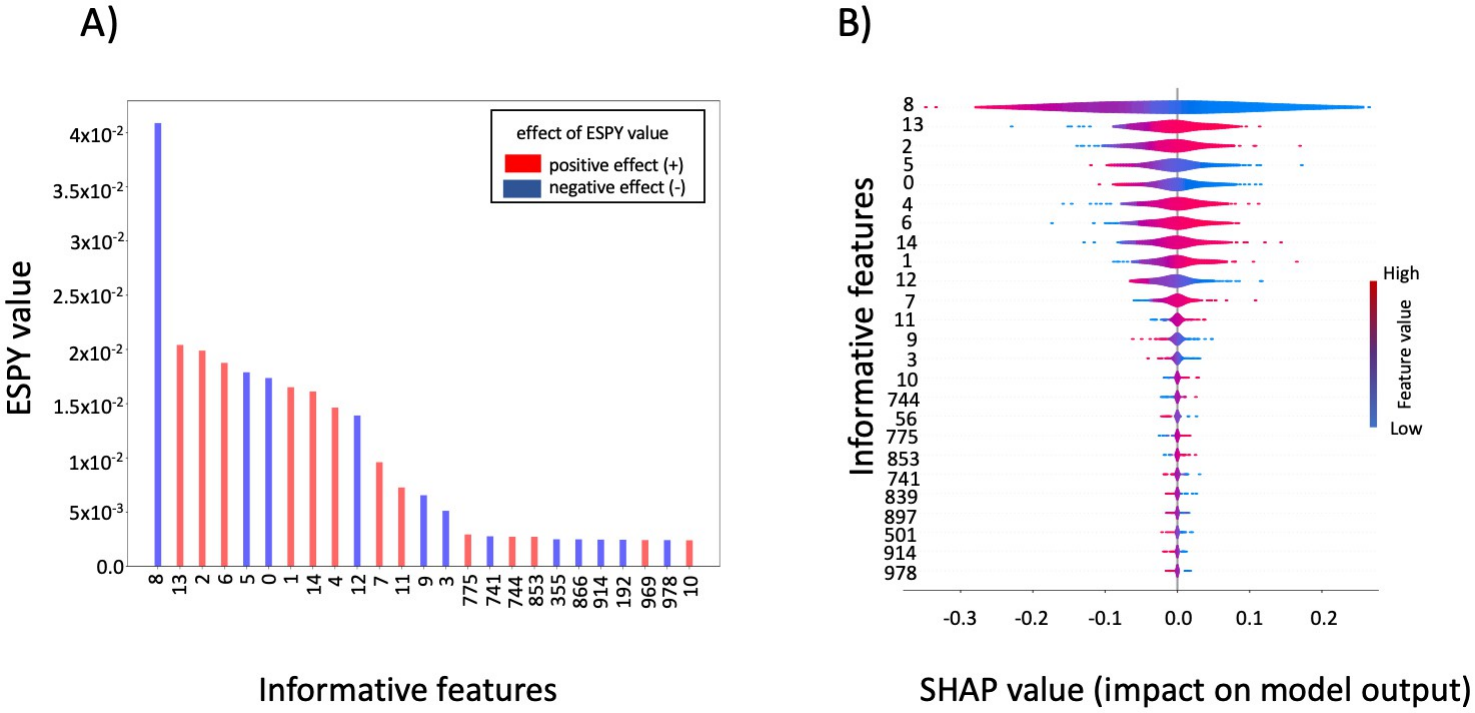
Informative features selected using ESPY and SHAP values on the simulated data. We show the top 25 features of the simulated data that were evaluated to be informative by ESPY and SHAP. A) The first 14 out of 15 informative features have higher ESPY values in comparison to rest of the features. For each feature the effect with the positive class is indicated by the color of the bars: a negative effect with the positive class (negative ESPY effect) is indicated by blue color and positive effect with the positive class (positive ESPY effect) is indicated by red color. B) Also for the SHAP evaluation, the first 14 out of 15 informative features have the highest mean SHAP values and are thus sorted to the top of the shown violin summary plot. The SHAP value and the color coded original feature value are used to indicate the change of each feature in the model prediction towards the positive or negative class. A high SHAP value indicates a change towards the positive class and vice versa for a low SHAP value.

Fig 5A shows the informative features as identified by ESPY evaluation of a RBF-SVM trained on simulated data. As described in detail in the materials and methods section (Simulated data), the simulated data set consists of 500 samples and 1000 features, where 15 features are defined as informative features and the remaining ones as uninformative features. We used a SVM model with a RBF kernel to evaluate the ESPY values on simulated data. The RBF-SVM model achieved the highest AUC score for values of the regularization parameter *C* = 10 and the RBF kernel parameter *γ* = 0.001 (AUC of 0.92 during a stratified 5-fold grid-search cross-validation on the training dataset, and an AUC of 0.81 on the hold-out test dataset). The aforementioned parameter combination was then used to train a RBF-SVM model on the training dataset, which was used for ESPY evaluation afterwards. Since the ESPY evaluation is based on the computation of the distances from the classification boundary (refer to the materials and methods section), the procedure for the evaluation of informative features is the same for a single-task or a multitask SVM.

Fig 5A shows the top 25 informative features and their respective ESPY values. The first 14 features have higher ESPY values and are thus sorted from the left to the right of the shown bar plot. They are by definition the informative features in the simulated data. Only the fifteenth feature (of the top 15 informative features in the simulated data), labeled by *feature*10, had lower ESPY values and thus were shifted to the end of the list of the top 25 informative features. The remaining ten of 25 features had lower ESPY values and are by definition uninformative features of the simulated data.

We compared our ESPY method with the SHAP (SHapley Additive exPlanations) framework from Lundberg et al. [19]. Fig 5B shows the evaluated SHAP values. Again, the first 14 features have a higher mean SHAP value and are thus sorted to the top of the shown violin summary plot. Once again, the fifteenth feature (*feature*10) shows a lower mean SHAP value in comparison to the top 14 informative features. Comparing the class association (whether a feature is more similar to the positive class or vice versa), it can be seen in Fig 5 that, while the ranking by the absolute ESPY and SHAP values of several of the top 15 features is permuted, the evaluated associations of the individual top 15 features are similar. SHAP took more than 13 hours to evaluate the features on a Dell XPS 13 with Intel i7–10510U CPU, 4 Cores and 16GB of memory.

In comparison, ESPY evaluated the features in only 2.08 seconds on the same laptop. As mentioned by [20], a faster run-time of the SHAP framework could be achieved by clustering the background dataset (i.e., the training dataset) used to evaluate SHAP using k-means clustering with not too many cluster centers (e.g., *k* = 50). However, in scenarios as studied here, where the data has many more dimensions than samples (curse of dimensionality) (*n* > >*m*), k-means is unlikely to find a good clustering of the data. In summary, we could show that our ESPY method and the SHAP framework perform similarly on the task of informative feature, which refelcts a real world dataset. However, the SHAP framework needed much more computation time compared to our approach.

## Discussion

In this study, we used a Pf-specific proteome microarray covering about 91% of the entire Pf proteome to identify PfSPZ-CVac-induced antibody profiles. As earlier suggested by Felgner et al. and Trieu et al. [21, 22] and later shown by Obiero et al. and Wichers et al. [6, 23, 24], the immune response against human malaria is induced by a wide range of Pf-specific antigens. Here, we adapted supervised machine learning methods to identify predictive antibody profiles from immunized and non-immunized PfSPZ-CVac individuals.

Due to the large number of antibody signal intensities in comparison to the small number of individuals per time point, we set up a customized multitask SVM approach. Multitask SVM models are known to perform very well on prediction problems that profit from combining related data into a single model to increase the number of samples for the prediction task [14]. We compared our final multitask SVM approach with state-of-the-art machine learning approaches, namely regularized logistic regression (RLR), random forest (RF), and a single-task SVM model with a RBF kernel, after immunization and before CHMI. Overall, the RLR model, the single-task SVM model, and the RF approach, which were either trained on each single time point separately or on samples from both time points, showed a lower performance in comparison to our multitask SVM approach, where both time points were combined into a single prediction model.

Using our multitask SVM approach, we show how to profit from combining time- and dose-dependent data from multiple time points into a sole prediction model, whenever the number of samples at each single time point is small. Moreover, we show that our multitask SVM approach is less affected by highly correlated features over a range of Pearson correlation coefficients and achieves robust accuracy scores in comparison to the state-of-the-art approaches (Figs A and B in S1 Appendix). This might be of great interest in the biomedical context, where the number of available samples is often limited and strong correlations between features can be assumed. Here, in the analysis of a large proteome microarray (p = 7,455) was analyzed, we assumed strong correlations due to e.g., similar epitopes, between fragments representing one protein, and cross-reactivity [15]. Furthermore, we excluded the antibody responses after CHMI (post-CHMI) from our malaria vaccine efficacy prediction analysis and our implementation of our multitask SVM approach due to the reason that the control group underwent a CHMI at this time point as well.

For the evaluation of informative Pf-specific antigens from the multitask SVM approach, we decided to only consider only the first top 50 Pf-specific antigens with the highest ESPY values per time point. Using ESPY evaluation, we estimated the contribution of each single Pf-specific antigen towards the classification of protected vaccinees and non-protected vaccinees and controls. In this analysis strongly linearly correlated features do not have the same influence on the evaluation procedure as they might have it in the RLR and RF approach. With this analysis we could show that PfSPZ-CVac immunized protected individuals react against a broad spectrum of known and unknown Pf-specific antigens, such as PfEMP1, CSP, MSP2/4, LSA1, GLURP conserved (membrane) proteins of unknown function, and intra-cellular/trans-membrane proteins after immunization and before CHMI.

The antibody breadth of PfSPZ-CVac immunized individuals varied based on the received PfSPZ-CVac dose. The number of identified Pf-specific antigens showed an overall, medium to high antibody reactivity among individuals who received a low and medium dose of PfSPZ-CVac and in the control group. Assuming that this result is not induced due to a high background noise, we hypothesize that these antigens are recognized by humoral immunity of individuals that were never in contact with human malaria before. This might be explained through cross-reactivity. Murugan et al. [25] supports the idea that the repertoire before immunization is important for the generation of high affinity antibodies (in this case anti-CSP). Further analysis of the antibody profile before vaccination of those individuals who underwent a successful immunization is needed to determine whether a high abundance of antibodies correlates with a higher chance of vaccination. Protected individuals who received the highest dose of PfSPZ-CVac showed higher antibody reactivity against a comparatively small set of Pf-specific cell-surface antigens of the pre-erythrocytic and erythrocytic stage, like CSP, PfEMP1, MSP2/4, LSA1, GLURP and conserved Pf (membrane) proteins with unknown function. In agreement with these findings, Mordmüller et al. [8] showed that LSA1, MSP4, GLURP and conserved Pf proteins with unknown function were recognized by more than half of the protected individuals in the high-dose group, whereas, PfEMP1 was not recognized by at least 5/9 of the protected individuals in the high-dose group. Further, Mordmüller et al. [8] showed by ELISA that all of those protected individuals strongly reacted against CSP. Our analysis of Pf-specific antigen identification evaluated most of the informative proteins found by Mordmüller et al. [8], as well as new ones that seem to improve the prediction performance. Antibodies against CSP have significant functional activity in the protection against human malaria [8, 26], and CSP is a dominant antigen in the early (pre-erythrocytic) phase of the infection. However, the most advanced malaria vaccine candidates, RTS,S (Mosquirix) and R21/Matrix-M, confer only limited and short-lived protection against clinical malaria in the former one [27–29] and in the latter one the R21/Matrix-M candidate achieved the WHO-stated vaccine efficacy goal over 75% [30, 31] against Pf clinical malaria but only in one specific malaria endemic area. We hypothesize that sterile protection against human malaria induced through PfSPZ-CVac is not alone conferred by the humoral immunity, but rather is the result of both humoral and cellular immune responses to a number of different antigens. Recent findings from many clinical studies [7, 32–35], administering chemoattenuated PfSPZ or irradiated sporozoites, reproducibly confirm that PfSPZ-based vaccines induce an increase in cellular immune responses. CD4 T cells, CD8 T cells and *γ* *δ* T cells are supposed to be primary effectors in the elimination of parasite-infected hepatocytes. Especially memory CD8 T cells are associated with the direct killing of infected hepatocytes; at least in animal models of malaria [36]. However, these processes occur in the liver and are therefore difficult to study in humans. Malaria vaccine candidates, such as RTS,S (Mosquirix) or R21/Matrix-M, target one major surface protein and thereby are not able to induce highly potent cellular immune responses against infected hepatocytes. Thus, the additive effect of antibodies in the ensemble of a cellular immune response against sporozoites is likely to lead to robust sterile protection and is yet missing in single protein based vaccine attempts. Further analysis of T- and B-cell mediated immune response against human malaria is hence needed for the prediction of a succsessful immunization.

A clear limitation of this study is that proteins on the microarray are not in their natural context and may have different conformation and post-translational modifications [37]. Furthermore, the microarray chip contains different concentrations of Pf-specific antigens per spot resulting in the problem of optimal quantification of informative Pf-specific antigens. This might also account for the relatively small number of identified Pf-specific antigens that were related to the protection status. In addition, the biological life cycle of the parasite is complex and the metabolically active parasite resides mostly intra-cellularly in hepatocytes and erythrocytes, with only transient extracellular phases. Therefore, the response is likely to mainly target cell-surface expressed antigens of merozoites, the extracellular form of Pf, and parasite-antigens that are presented by the infected cell. Importantly, the small number of identified Pf-specific antigens were sufficient to discriminate the protection status of protected vaccinees and non-protected vaccinees and controls. In general, all methods we have used are limited, if the antibody profiles are not similar between subjects. Here, the antibody breadth might be more important than a specific pattern favoring sterile protection against human malaria.

## Conclusion

The large number of more than 5,300 genes that are expressed during the life cycle of Pf, may explain why we still do not know which antigens are central to the induction of a sterile protection against human malaria. Proteome microarrays enabled us to measure antibody reactivity against Pf-specific antigens representing about 91% of the Pf proteome. Felgner et al., Obiero et al., and Illingworth et al. [6, 21, 38] earlier suggested that the immune response against human malaria is induced by a wide range of these proteins, yet only a few of these proteins have been clinically tested as a malaria vaccine. In medical trial studies, where new anti-malaria vaccine candidates are tested, the number of samples is often restricted to a small size. To overcome this problem, here we combined time- and dose-dependent data of PfSPZ antibody profiles of immunized and non-immunized individuals from multiple time-points into one sole prediction model. This approach is beneficial, since clinical vaccine studies are usually limited in their number of samples. Additionally, we proposed the new ESPY method to explain predictions from a non-linear SVM model. We could show, on simulated data, that ESPY evaluation can identify all informative features and provides explanations comparable to the SHAP framework for kernel SVMs. We successfully applied the ESPY method to find informative Pf-specific antigens for the prediction of protected and non-protected PfSPZ-CVac vaccinees and controls based on their antibody reactivity profiles. Our findings might help to extend the knowledge about Pf-specific antigens that induce B-cell activation. However, to fully understand the immune response against human malaria, a further step will be to include T-cell activation and RNA-seq data.

## Materials and methods

### Ethics statement

The study was approved by the ethics committee of the medical faculty and the university clinics of the University of Tübingen (project number 537/2013AMG1). All trial participants were thoroughly informed and gave written informed consent before any study procedure, recording of data or analysis was carried out.

### Data

#### Proteome microarray

We analyzed the Pf-specific antibody reactivity profile from the PfSPZ-CVac clinical trial TÜCHMI-002 (ClinicalTrials.gov Identifier: NCT02115516), which has been previously described [8]. In brief, Pf-specific antibody-mediated response profiles of 40 malaria-naïve individuals, vaccinated three times over 8 weeks with placebo (normal saline) or different doses of PfSPZ (3.2 × 10^3^ PfSPZ, 1.28 × 10^4^ PfSPZ, 5.12 × 10^4^ PfSPZ) by direct venous inoculation under chloroquine chemoprophylaxis (Sanaria PfSPZ-CVac), were measured at four different time points: before vaccination (I-1), following the third vaccination (III+14), one day before CHMI (C-1), and four weeks after CHMI (C+28). The proteome microarray contains 7,455 Pf-specific protein fragments representing 4,805 unique Pf genes of the NF54 Pf strain. This resulted in a dataset containing 40 individuals per time point with Pf-specific antibody intensity signals from 7,455 Pf-specific fragments. At each dosage, 9 individuals were vaccinated (for a total of 27 across 3 PfSPZ-CVac doses) and 13 individuals were allocated to the placebo group. For each individual, the protection status is defined by the primary efficacy endpoint as described earlier by Mordmüller et al. [8]. An individual was considered not protected against malaria, if any parasitemia was detected by thick blood smear, and protected, if no parasite was detected within 21 days following CHMI. All parasitemic volunteers were treated promptly with a highly active antimalarial. The data underlying this study were obtained from proteome microarray data as described by Mordmüller et al. [8]: “Raw spot and local background fluorescence intensities, spot annotations and sample phenotypes were imported and merged in R, where all subsequent procedures were performed. Foreground spot intensities were adjusted by local background by subtraction, […].” Baseline (I-1) antibody responses of immunized and control individuals were subtracted from the data generated post-immunization (III+14 and C-1) for each individual to focus on PfSPZ-CVac induced antibody responses [6]. Antibody responses after CHMI (C+28) were excluded from our malaria vaccine efficacy prediction analysis, because also controls underwent CHMI at this time point. Thus, after CHMI, there are no samples for the unprotected class anymore and, therefore, applying binary classification models will not be feasible. The resulting dataset contains 80 samples associated to 40 patients at two different time points (III+14 and C-1). Subsequently, the Pf-specific antibody signal intensities were arcsine transformed.

In a second step, we defined a set of cell surface Pf-antigen fragments from the whole set of Pf-specific fragments of the proteome microarray. Cell surface Pf-antigen fragments and Pf-antigen fragments of uncharacterized proteins (m = 1,194) were selected from the proteome microarray supplement information based on their protein name/description, representing extracellular/membrane and uncharacterized proteins. In this subset, we assumed that responses to cell surface antigens are over-represented compared to intra-cellular antigens.

#### Simulated data

To evaluate how our feature importance measurement performs, we used python and *sklearn.datasets.make_classification(n_samples = 500, n_features = 1000, n_informative = 15, n_redundant = 0, n_repeated = 0, n_classes = 2, random_state = 42)* from the *Scikit-learn* [39] package to generate a random two-class classification problem. Said method creates clusters of points that are standard normally distributed about vertices of an 15-dimensional hyper-cube and assigns an equal number of clusters to each class. The resulting data matrix $X\in R^{500\times 1000}$ consists of 15 informative feature columns, while the remaining feature columns are filled with random noise.

### Prediction models for time-series data

Our proposed approach for identifying Pf-specific immune signatures from protected and non-protected individuals tackles two problems. First, identifying appropriate machine learning models that are able both to deal with high-dimensional data and can learn from time and dose dependent data. Second, identifying informative Pf-specific antigens between protected and non-protected individuals.

Since individuals were exposed to different PfSPZ doses in the PfSPZ-CVac clinical trial at two (actually three, but we excluded the third time point, due to the reasons described in section Proteome microarray) consecutive time points, we built different prediction models to analyze the dependent time-series data. All models are binary classifiers that predict the protection state of the Pf-specific proteome microarray-based on antibody reactivity profile.

In a first scenario, the RLR [11], RF, and single-task RBF-SVM models were trained separately for both time-points. For this purpose, the dataset was split into two smaller “single-time” datasets corresponding to the time points III+14 and C-1, respectively. Each dataset contains antibody intensity signals for all PfSPZ doses (3.2 × 10^3^ PfSPZ, 1.28 × 10^4^ PfSPZ, 5.12 × 10^4^ PfSPZ), associated to a single time point, augmented by the PfSPZ dose as auxiliary information.

Tasks, that are related to each other, can be used in a prediction model simultaneously (so-called *multitask-learning*) [40, 41]. Considering the small number of samples per time point and the very large amount of features (sample size *N*_patients_ = 40, number of features *P* = 7, 455), establishing a prediction model that uses related samples from additional time points is more promising than training independent models with data for the specific task only. Therefore, in a second scenario, we cast the prediction problem into a multitask learning problem and treat prediction based on time point, PfSPZ dose, and antibody intensity signal data as separate tasks. To implement the multitask approach, we represented the relationships between individuals for each task (time point, PfSPZ dose, antibody intensity signals) by a separate feature matrix. Within the multitask approach we were able to classify individuals into protected and non-protected using the measured immune profile of all 40 individuals for each time point (III+14 and C-1) and dose (3.2 × 10^3^ PfSPZ, 1.28 × 10^4^ PfSPZ, 5.12 × 10^4^ PfSPZ) at once in one model resulting in 80 samples.

In a third scenario, to accomplish a fair comparison between our multitask SVM approach and the other methods, namely RLR, RF and RBF-SVM, we additionally trained those on the original “multi-time” dataset, containing 80 samples associated to 40 patients at two different time points (III+14 and C-1) augmented by the PfSPZ dose. For details please refer to the subsequent section Prediction performance assessment.

### A multitask SVM approach for time- and dose-dependent proteome data

Kernels of SVMs can be used to model relationships between single related tasks and combine them into one prediction model [42, 43]. Combining such small single datasets, that are related to each other, can lead to an improvement in classification. Kernel-based multitask learning can be achieved by the element-wise product of two kernel matrices. According to the *Schur product theorem* [44], the Hadamard (element-wise) product of two positive (semi-)definite matrices is also a positive (semi-)definite matrix. For the measured immune profile, an antibody signal intensity matrix *X*_antibody_ ∈ $R^{N\times P}$ is given, where *N* is the number of samples (*N* = 2 × *N*_patients_) and *P* the number of features. The value at entry *x*_*np*_ is the detected antibody signal intensity of feature *p* for sample *n*. Additionally, two vectors are given: the time-series vector $\gamma_{\text{t}}\in R^{N}$, where *t* represents the date of antibody profile collection, and the dose vector $\gamma_{\text{d}}\in R^{N}$, where *d* represents the dose of PfSPZ. To simulate the clinical trial of PfSPZ-CVac over the two time points, we used a radial basis function (RBF) kernel to represent the relationship between the individuals based on time points, resulting in the kernel matrix *K*_1_(*n*_*t*_, *n*_*t*′_). As a representation of the relationship between the individuals based on the administered PfSPZ dose, we used either an RBF or a polynomial kernel function, resulting in a kernel matrix *K*_2_(*n*_*d*_, *n*_*d*′_). Finally, the relationship between the individuals based on antibody signal intensities was represented by either an RBF or a polynomial kernel function, resulting in a kernel matrix *K*_3_(*n*_*p*_, *n*_*p*′_). The resulting kernel matrices were combined by element-wise multiplication:

$$\begin{array}{c} K_{\text{multitask}}\left(\left(n_{t},n_{d},n_{p}\right),\left(n_{p^{\prime }},n_{t^{\prime }},n_{p^{\prime }}\right)\right)≔\\ \;K_{1}\left(n_{t},n_{t^{\prime }}\right)\times K_{2}\left(n_{d},n_{d^{\prime }}\right)\times K_{3}\left(n_{p},n_{p^{\prime }}\right) \end{array}$$ (1)

where *K*_multitask_ is a positive semi-definite (psd) kernel matrix, containing all feature intensities signals for two time points (III+14 and C-1) and all PfSPZ doses.

### Adapted spectral translation approach

The *Schur product theorem* [44] states that the Hadamard product of two psd matrices is psd as well. In practice, due to numerical issues arising from limited machine precision, the multitask kernel matrix *K*_multitask_, resulting from the element-wise multiplication of the single-task kernel matrices, might be slightly disturbed and not psd within numerical precision. To deal with this issue, we implemented the following procedure.

After element-wise multiplication of the single-task kernel matrices, we applied a variant of the so-called *spectral translation approach* [45]. Originally, as described by Vert et al. [45], the smallest negative eigenvalue is subtracted from (i.e., its absolute value is added to) the diagonal of a non-psd symmetric matrix, resulting in a psd kernel matrix. This approach exploits the fact that a psd matrix is symmetric and all its eigenvalues are non-negative per definition. By adding the absolute value of the smallest negative eigenvalue to its diagonal, the spectrum of the non-psd matrix is effectively shifted upwards and, thus, the matrix becomes psd. Since, in practice, again due to numerical instabilities, this approach sometimes does not result in a psd matrix, we adapted it into an iterative procedure as follows: We computed the real eigenvalues *e*_*i*_ of *K*_multitask_ and tested if any of them were negative. If negative eigenvalues were found, $\text{max}\left(\left\{\tilde{\epsilon },\left|{\text{min}}_{i}\left(e_{i}\right)\right|\right\}\right)$ was added to the diagonal of *K*_multitask_, with $\tilde{\epsilon }=10^{3}\cdot \epsilon$ and *ϵ* being the machine precision. This approach was repeated for a maximum of 1000 iterations (if the matrix was not psd after 1000 iterations, a value of 1.0 was added to the diagonal and the resulting matrix was used) or until no further negative eigenvalues were found, thus resulting in a psd multitask kernel matrix *K*_multitask_. Based on the multitask kernel matrix, we trained and tested different settings of parameters to create a predictive model.

### Dealing with strongly linearly correlated features

To better understand and predict a successful vaccination, identification and interpretation of single Pf-specific antigens is important. In the underlying dataset we are dealing with a large number of features compared to a small number of samples. Further, strong correlations between the features can be assumed due to, e.g., several fragments representing a single protein, similar epitopes, and cross-reactivity [15]. Reducing the number of irrelevant and uninformative features can increase performance [13, 16, 17]. As earlier shown by Valletta et al. [13], a Pearson correlation coefficient of *pcc* = 0.8 seems to be a reasonable threshold for removing strongly linearly correlated features of such proteome microarray data. Nonetheless, we assessed the influence of correlated features on the prediction performance of the studied machine learning models, by removing features that were linearly correlated above a certain Pearson correlation threshold *pcc*, varying the threshold between 0.1 and 1.0 in steps of 0.1. The results of this analysis are visualized in supplementary figures Figs A and B in S1 Appendix. For both datasets, the whole proteome microarray (Fig A in S1 Appendix) and the pre-selected cell surface microarray (Fig B in S1 Appendix), we can show, that the prediction performance of the state-of-the-art methods is more influenced by strongly linear correlated features than our multitask SVM approach with a kernel combination of either ‘RRR’ or ‘RPR’. Furthermore, the Pearson correlation coefficient of *pcc* = 0.8 seems to be also a reasonable threshold for our datasets. Therefore, we removed strongly linear correlated features above a Pearson correlation coefficient of *pcc* = 0.8, which corresponds to around 8% of the original whole proteome microarray and to around 16% of the original pre-selected cell surface microarray.

### Prediction performance assessment

To assess the prediction performance of the studied machine learning models, we proceeded as follows. We compared multitask SVM models with single- and multi-time single-task RBF-SVM, RF, and RLR models.

For the RLR models we used the so-called *elastic net* regularization as implemented in the *sklearn*.*linear*_*model*.*LogisticRegression* class of the *Scikit-learn* [46] package. Elastic net RLR uses a combination of *ℓ*_1_ and *ℓ*_2_- regularization, where *ρ* defines the compromise between *ℓ*_1_ (*ρ* = 1) and *ℓ*_2_ (*ρ* = 0) penalty, and minimizes the following cost function:


$$\begin{array}{c} \underset{w,c}{\text{min}}\;(\frac{1-\rho }{2}w^{T}w+{\rho \|w\|}_{1}+C\sum_{i=1}^{N}\text{log}\left(\text{exp}\left(-y_{i}\left(X_{i}^{T}w+c\right)\right)+1\right)) \end{array}$$ (2)

We tuned and assessed the single- and multi-time RLR models during a 10-times repeated nested stratified 5-fold cross-validation, as described by Krstajic (Algorithm 2 in [47]) and implemented in the *RepeatedStratifiedNestedCV* class of the *nestedcv* [48] package, over a grid of *ρ* and the regularization parameter *C* (Table A in S1 Appendix). The single- and multi-time single-task RBF-SVM models (using the C-Support Vector Classification method in *sklearn*.*svm*.*SVC*) were tuned and assessed likewise by performing a 10-times repeated nested stratified 5-fold cross-validation over a grid of the RBF kernel coefficient *γ* and regularization parameter *C* (Table A in S1 Appendix). In case of single- and multi-time RF models, we used the *RandomForestClassifier* implemented in *sklearn*.*ensemble* and assessed its performance likewise by executing a 10-times repeated nested stratified 5-fold cross-validation over a number of parameters, namely the number of trees *n*_trees_ and the maximal number of features max_features per split (Table A in S1 Appendix). The multitask SVM models were deployed by pre-computing the multitask kernel matrices as described above in section A multitask SVM approach for time- and dose-dependent proteome data for different parameter combinations (listed in Table A in S1 Appendix) and feeding the pre-computed kernel matrices into the C-Support Vector Classification method implemented in *sklearn*.*svm*.*SVC*. The models were assessed and optimized over a parameter grid (listed in Table A in S1 Appendix) using 10-times repeated nested stratified 5-fold cross-validation utilizing *nestedcv*.*RepeatedStratifiedNestedCV*. All models were optimized and evaluated on exactly the same cross-validation folds. In case of multitask SVM models and multi-time RF, RLR, and single-task RBF-SVM models, test sets (aka test “folds” in the cross-validation context) were always constituted of samples from a certain single time point (III+14 or C-1), while the associated train folds were constituted from samples from both time points (III+14 and C-1). It was ensured that all samples taken from patients that were represented by a sample included in a test fold were excluded from the associated disjoint train fold. Model performances were measured using the *Area Under the Precision Recall Curve* (PR-AUC) where a higher PR-AUC score equates to perfect prediction and a lower PR-AUC score to random guessing. Precision recall curves are commonly used to evaluate the prediction performance of a model with imbalanced data, that is, when the number of samples of one class (e.g. class 0) is much higher than the number of samples of the other class (e.g. class 1) [49]. To assess the prediction performance of the underlying models, namely RLR, single-task RBF-SVM, RF and multitask SVM models, we performed 10 repetitions of nested stratified 5-fold cross-validation for each model, as described in detail above. To evaluate the PR-AUC, the predictions on the test sets of the models trained on the train folds were collected and used to calculate a nested cross-validation PR-AUC score for every repetition as described by Krstajic (Algorithm 2 in [47]). The final nested cross-validation score is then calculated as the average of the resulting scores of each repetition.

### Tuning models for feature evaluation

Before using the multitask SVM approach to evaluate informative features, we had to optimize the parameters. The multitask SVM models were deployed as described in the previous section. They were tuned over a parameter grid (listed in Table A in S1 Appendix) using 10-times repeated stratified 5-fold grid-search cross-validation, as described by Krstajic (Algorithm 1 in [47]), utilizing *nestedcv*.*RepeatedGridSearchCV*. Again, all models were optimized and evaluated on exactly the same cross-validation folds. In the case of multitask SVM models, test folds always comprised of samples from a certain single time point (III+14 or C-1), while the associated train folds comprised samples of both time points (III+14 and C-1). It was ensured that all samples taken from patients that were represented by a sample included in a test fold were excluded from the associated disjoint train fold.

The obtained parameters were then used to predict the mean PR-AUC performance on the hold-out test dataset for the whole proteome microarray and the pre-selected cell-surface proteome microarray and are listed in Table B in S1 Appendix. Afterwards, the trained model (either trained on the whole proteome microarray or on the pre-selected cell-surface proteome microarray) was used to evaluate the ESPY value (as explained in detail in the following section ESPY (fEature diStance exPlainabilitY)) for each single feature on the test data.

### ESPY (fEature diStance exPlainabilitY)

We propose ESPY values as a measure of importance of each single feature based on a (multitask) SVM model. The method is inspired by a feature importance measure for sequence-based non-linear predictions proposed by Pfeifer and Lengauer [18]. ESPY uses systematically and specifically triggered changes in the distance of a consensus sample to the classification boundary of the SVM to estimate the importance of features as described below. The distance to the classification boundary can be computed for a sample $\vec{x}$, the optimized parameter *α* ∈ *R*^*n*^, offset *b*, and class labels *y*_*i*_ ∈ [0, 1]:


$$\begin{array}{c} d\left(\vec{x}\right)=\sum_{i=1}^{n}y_{i}\left(\alpha_{i}k\left(\vec{x},{\vec{x}}_{i}\right)+b\right) \end{array}$$ (3)

The idea of the approach is to determine if a change in the antibody intensity signal of each feature at a time results in a distance change towards the negative or positive side of the classification boundary. If the signal leads to a change to the positive side, the antibody response is more similar to the positive samples and vice versa. To compare the change in the antibody intensity signal of each feature at a time, a consensus sample is generated. The consensus sample ${\vec{x}}^{\;Q_{2}}$ is element-wise defined as the median (alias second quartile *Q*_2_) antibody intensity signal $x_{j}^{\;Q_{2}}=\text{median}\left(X_{j}\right)$ over all samples per feature *X*_*j*_. *X*_*j*_ is the j-th column of the data matrix $X\in R^{N\times M}$, which was constructed by appending the time point vector $\gamma_{\text{t}}\in R^{N}$ and the dose vector $\gamma_{\text{d}}\in R^{N}$ as columns to the antibody signal intensity matrix *X*_antibody_ ∈ $R^{N\times P}$. To evaluate the ESPY value and therefore the importance of each feature, we measured the change in distance when varying the consensus at a certain feature *j* to the first quartile *Q*_1_ (25% percentile) and third quartile *Q*_3_ (75% percentile), respectively:

$$d_{j}^{Q_{1}}=d\left({\vec{x}}_{\;j}^{\;Q_{1}}\right)-d\left({\vec{x}}^{\;Q_{2}}\right)$$ (4)

$$d_{j}^{Q_{3}}=d\left({\vec{x}}_{\;j}^{\;Q_{3}}\right)-d\left({\vec{x}}^{\;Q_{2}}\right)$$ (5)

where ${\vec{x}}_{j}^{Q_{p}}≔(x_{1}^{\;Q_{2}},x_{2}^{\;Q_{2}},...,x_{j-1}^{\;Q_{2}},x_{j}^{Q_{p}},x_{j+1}^{\;Q_{2}},...x_{m}^{\;Q_{2}}{)}^{T}$ and $x_{j}^{Q_{p}}$ is the *p*th quartile of *X*_*j*_. Based on this, we define the ESPY value per feature *j* as:

$$\begin{array}{c} I_{\text{ESPY},j}≔|d_{j}^{Q_{1}}\left|+\right|d_{j}^{Q_{3}}|\;, \end{array}$$ (6)

$$\begin{array}{c} M_{\text{ESPY},j}≔\{\begin{array}{cc} + & ,\;d_{j}^{Q_{3}}\geq 0\;\wedge \;d_{j}^{Q_{1}}\leq 0\wedge d_{j}^{Q_{3}}\neq d_{j}^{Q_{1}}\\ - & ,\;d_{j}^{Q_{3}}\leq 0\;\wedge \;d_{j}^{Q_{1}}\geq 0\wedge d_{j}^{Q_{3}}\neq d_{j}^{Q_{1}}\\ 0 & ,\;d_{j}^{Q_{3}}=d_{j}^{Q_{1}}=0\\ \text{NAN} & ,\;\text{else} \end{array}\;, \end{array}$$ (7)

with +, −, 0, and NAN denoting a *positive effect*, *negative effect*, *no effect*, and an *undefined effect* respectively. Finally, the ESPY value per feature *j* is normalized by dividing each ESPY value by the sum of the ESPY values of all features.

#### ESPY evaluation on simulated data

To evaluate the ESPY values of each feature in the simulated dataset based on 7, we trained an RBF-SVM model. At first, the simulated dataset was split into training and test sets (70% for training, 30% for testing). This was followed by hyper-parameter tuning using stratified 5-fold grid-search cross-validation (as implemented in *sklearn*.*model*_*selection*.*GridSearchCV*) over a grid of the RBF *γ* parameter and the regularization parameter *C* (see Table A in S1 Appendix) on the training data, to evaluate the best parameter combination for the RBF-SVM model. The obtained parameters were then used to predict the AUC performance on the hold-out test dataset. Afterwards, the trained model was used to evaluate the ESPY value for each single feature on the test data.

We compared our explanation and feature extraction method, namely ESPY, with the SHapley Additive exPlanations (SHAP) method [19], a game-theoretic approach for making any machine learning model interpretable. Particularly, we applied the *shap*.*KernelExplainer* class of the *shap* [50] package on the trained RBF-SVM model and evaluated the features with the highest importance scores.

## Supporting information

S1 Appendix This PDF file compiles supplementary information of this paper. (PDF)
