# Supplementary material for: Machine learning prediction of malaria vaccine efficacy based on antibody profiles
Source: PLoS Comput Biol. 2024 Jun 7;20(6):e1012131. doi: 10.1371/journal.pcbi.1012131 (PMC11189177; doi:10.1371/journal.pcbi.1012131)
Supplement: S1 Appendix — (PDF) [file pcbi.1012131.s001.pdf]

Machine Learning Prediction of Malaria Vaccine Efficacy based on Antibody Profiles

Supplementary Data

Jacqueline Wistuba-Hamprecht, Bernhard Reuter, Rolf Fendel, Stephen L. Hoffman, Joseph J.Campo, Phil L. Felgner, Peter G. Kremsner, Benjamin Mordmüller, Nico Pfeifer

**SVM:**	**Parameter**	from	to	by
**Kernel**				
polynomial	degree	2	6	1
rbf	gamma	10^{-6}	10^{6}	1
cost parameter	C	10^{-4}	10^{5}	1
**RLR:**				
	C	10^{-4}	10^{4}	1
	rho	0	1	0.1
**RF:**				
	n_trees	100	1000	500
	max_features	['sqrt', 0.1, 0.333]		

Table A: Parameter ranges for evaluation of the best combinations of kernels for the multitask SVM approach. Parameter ranges for the ten time repeated nested stratified 5-fold cross-validation of the RLR, RF and single-task SVM (with rbf kernel) methods.

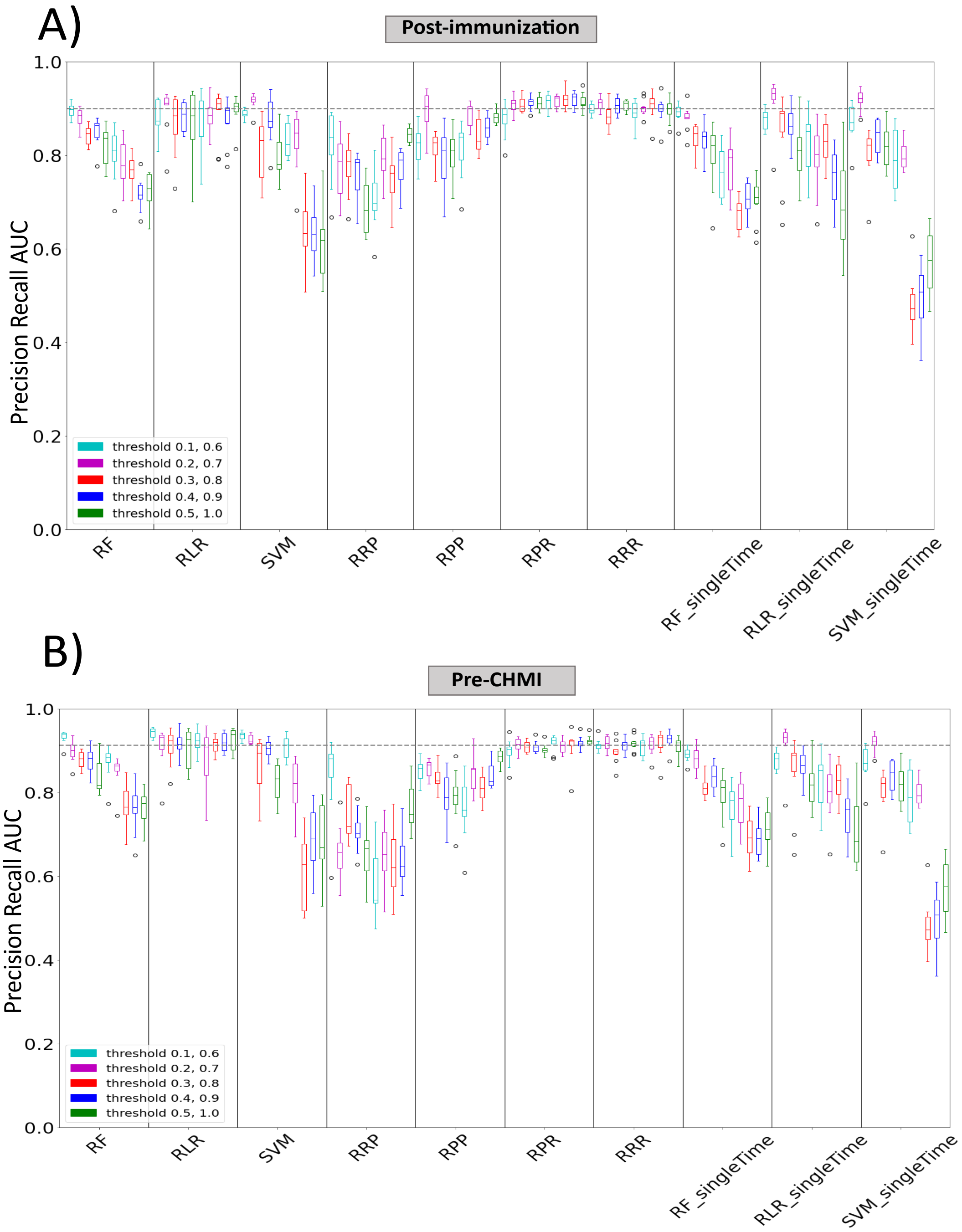

Fig A: Performance of multitask SVM models in in predicting the protection status based on the antibody reactivity profile over different Pearson correlation coefficients as compared to state-of-the-art approaches. The PR-AUC score of the RLR, the RF, the single-task SVM (trained either on each single time point or on the combined time points), and the multitask-SVM models (using different combinations of kernel functions) for predicting the protection status based on the whole proteome antibody profile over a range of Pearson correlation coefficients from 0.1 to 1 was assessed via 10-times repeated nested stratified 5-fold cross-validation. RF, RLR and single-task SVM models trained on each time point separately are labeled by the extension 'singleTime'. The PR-AUC performance of the different applied models is shown **A:** at post-immunization (III+14) and **B:** at pre-CHMI (C-1). The multitask-SVM approach with a kernel combination of either ‘RPR’ or ‘RRR’ shows a robust PR-AUC score over the range of Pearson correlation coefficients compared to the state-of-the-art approaches at post-immunization **(A)** and at pre-CHMI **(B).** Only multi-time RLR models achieved similar prediction performances over the range of Pearson correlation coefficients, but with a higher standard deviation at post-immunization **(A)** and at pre-CHMI **(B)**. The dashed line represents the mean PR-AUC over all Pearson correlation coefficients for the multitask-SVM model with the kernel combination of ‘RRR’.
A: The mean PR-AUC score over all Pearson correlation coefficients of the multitask-SVM approach with a kernel combination of ‘RRR’ is 0.899 and higher than the mean PR-AUC score of the multi-time RLR approach of 0.880.
B: The multitask-SVM approach with a kernel combination of ‘RRR’ and the multi-time RLR approach achieved the same mean PR-AUC score over all Pearson correlation coefficients of 0.914.

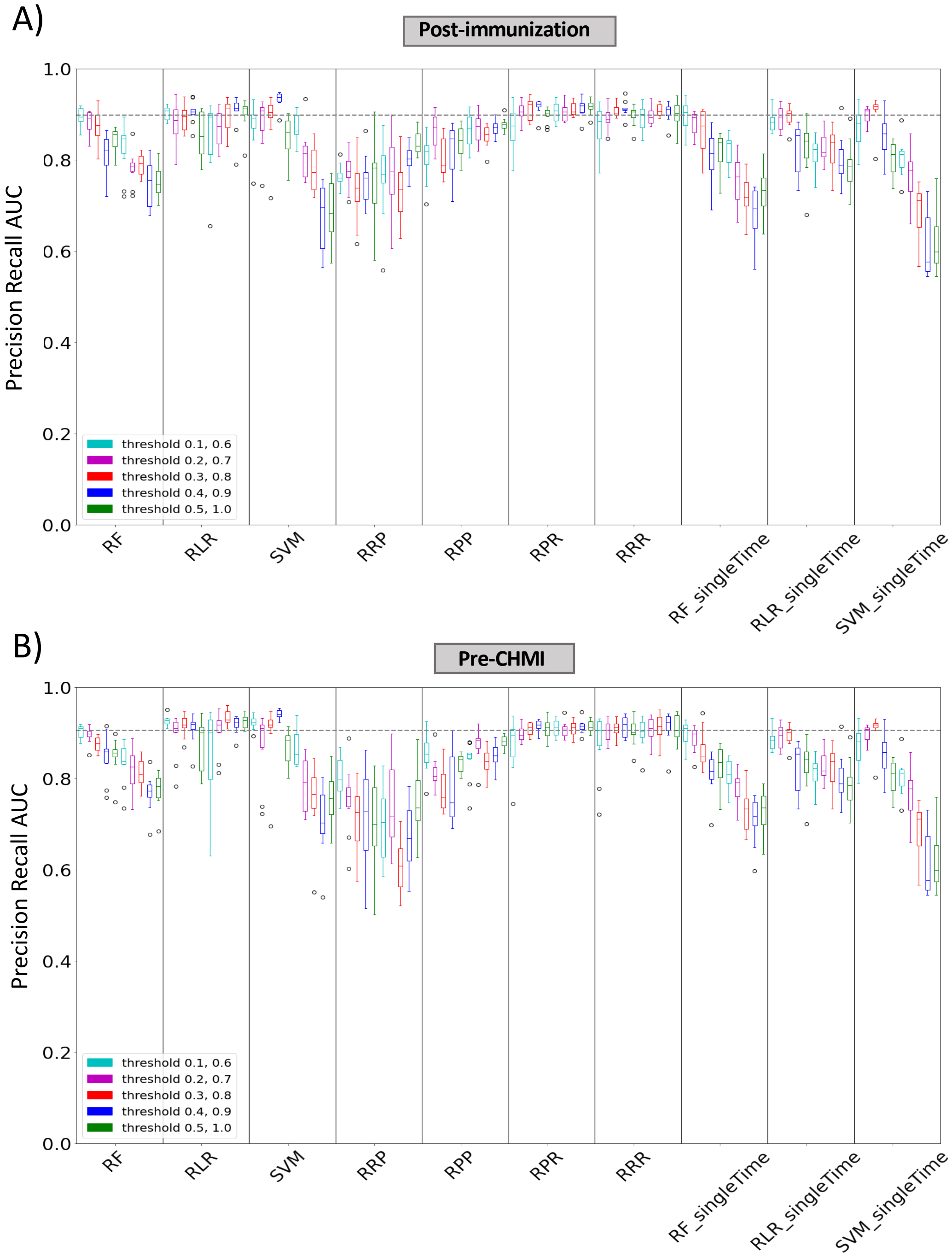

Fig B: Performance of multitask SVM models in in predicting the protection status based on cell-surface antibody reactivity profile over different Pearson correlation coefficients as compared to state-of-the-art approaches. The PR-AUC score of the RLR, the RF, the single-task SVM (trained either on each single time point or on the combined time points), and the multitask-SVM models (using different combinations of kernel functions) for predicting the protection status based on cell-surface antibody reactivity profile over a range of Pearson correlation coefficients from 0.1 to 1 was assessed via 10-times repeated nested stratified 5-fold cross-validation. RF, RLR and single-task SVM models trained on each time point separately are labeled by the extension 'singleTime'. The PR-AUC performance of the different applied models is shown **A:** at post-immunization (III+14) and **B:** at pre-CHMI (C-1). The multitask-SVM approach with a kernel combination of either ‘RPR’ or ‘RRR’ shows a robust PR-AUC score over the range of Pearson correlation coefficients compared to the state-of-the-art approaches at post-immunization **(A)** and at pre-CHMI **(B).** Only multi-time RLR achieved similar prediction performances over the range of Pearson correlation coefficients, but with a higher standard deviation at post-immunization **(A)** and at pre-CHMI **(B)**. The dashed line represents the mean PR-AUC score over all Pearson correlation coefficients for the multitask-SVM model with the kernel combination of ‘RRR’.

A: The mean PR-AUC score over all Pearson correlation coefficients of the multitask-SVM approach with a kernel combination of ‘RRR’ is 0.898 and higher than the mean PR-AUC score of the multi-time RLR approach of 0.885.

B: The mean PR-AUC score over all Pearson correlation coefficients of the multitask-SVM approach with a kernel combination of ‘RRR’ is 0.905 and disproportionally lower than the mean PR-AUC score of the multi-time RLR approach of 0.906.

Data Set	Time Point	Combination of kernel functions for time-series, Pf-SPZ dose and Ab signal intensities	Combination of kernel parameter	mean PR-AUC score +/- std
Whole protoeme data (m=40 samples, p=7,455 features)	III+14 (post-immunization)	RRR	R0: 0.1<br>R1: 0.01<br>R2: 1e-06<br>C: 1.0	0.927<br>+/- 0.009
Whole protoeme data (m=40 samples, p=7,455 features)	C-1 (pre-CHMI)	RRR	R0: 0.1<br>R1: 0.1<br>R2: 1e-05<br>C: 0.1	0.943<br>+/- 0.006
Selective set of cell-surface antigens (m=40, p=1194 features)	III+14 (post-immunization)	RRR	R0: 1.0<br>R1: 0.1<br>R2: 1e-05<br>C: 1.0	0.932<br>+/- 0.013
Selective set of cell-surface antigens (m=40, p=1194 features)	C-1 (pre-CHMI)	RRR	R0: 0.1<br>R1: 0.1<br>R2: 1e-05<br>C: 0.1	0.942<br>+/- 0.011
Whole protoeme data (m=40 samples, p=7,455 features)	III+14 (post-immunization)	RPR	R0: 1.0<br>P1: 3<br>R2: 1e-05<br>C: 0.01	0.0929<br>+/- 0.009
Whole protoeme data (m=40 samples, p=7,455 features)	C-1 (pre-CHMI)	RPR	R0: 10<br>P1: 2<br>R2: 1e-05<br>C: 0.01	0.936<br>+/-0.009
Selective set of cell-surface antigens (m=40, p=1194 features)	III+14 (post-immunization)	RPR	R0: 1e-06<br>P1:2<br>R2: 1e-05<br>C:0.1	0.936<br>+/- 0.006
Selective set of cell-surface antigens (m=40, p=1194 features)	C-1 (pre-CHMI)	RPR	R0: 0.01<br>P1: 2<br>R2: 1e-05<br>C: 0.01	0.933<br>+/- 0.016

Table B: Performance results of 10 times repeated 5-fold grid search CV for different combination of kernel combination and their parameter setting of the multitask SVM based on the whole and selective set of cell-surface antibody reactivity profile per time point. To evaluate the best prediction performances (PR-AUC) for the different kernel combinations for the kernel parameter ranges from **S1 Table** a 10 time repeated 5-fold grid search CV on the whole antibody reactivity profile and the pre-selected cell-surface antigens from immunized and non-immunized individuals were performed. The table shows the kernel combinations and their parameter setting which resulted in the highest mean PR-AUC per time point. R0 represents the gamma value of the rbf kernel function for the time series, R1 or P1 represents either the value of the rbf kernel function or the polynomial kernel function for the Pf-SPZ dose and R2 for the antibody signal intensity. Based on the highest mean PR-AUC value of the kernel combination per time point (III+14 or C-1) , the kernel parameter combination 'RRR' was used to evaluate the ESPY value for each feature in the multitask-SVM classification of protected immunized and non-protected immunized/non-immunized individuals based on the whole and selective cell-surface antigen antibody reactivity profile.

Plasmodb ID	effect	ESPY value (|d_norm|)	Description
PFSPZ dosis	+	6.26E-01	PFSPZ dosis
PF3D7_1123200	+	1.45E-03	leucine-rich repeat protein
PF3D7_0210200	+	1.03E-03	conserved Plasmodium protein, unknown function
PF3D7_0628100	+	9.49E-04	HECT domain-containing protein 1, putative
PF3D7_0526200	+	9.32E-04	ADP-ribosylation factor GTPase-activating protein, putative
PF3D7_1138400	+	7.29E-04	guanylyl cyclase (GCalpha)
PF3D7_0815500	+	7.17E-04	conserved Plasmodium protein, unknown function
PF3D7_1032900	+	6.51E-04	RNA polymerase II-associated protein 1, putative
PF3D7_0902200	+	5.91E-04	serine/threonine protein kinase, FIKK family (FIKK9.3)
PF3D7_1364400	+	5.20E-04	conserved Plasmodium protein, unknown function
PF3D7_1332900	+	5.19E-04	isoleucine-tRNA ligase, putative
PF3D7_1008000	+	5.08E-04	histone deacetylase 2
PF3D7_0804500	+	4.85E-04	conserved Plasmodium membrane protein, unknown function
PF3D7_1227200	+	4.74E-04	potassium channel K1
PF3D7_1329500	+	4.67E-04	conserved Plasmodium protein, unknown function
PF3D7_1243900	+	4.58E-04	double C2-like domain-containing protein
PF3D7_0807100	+	4.29E-04	DNA helicase PSH3
PFC0210c	+	3.91E-04	circumsporozoite (CS) protein (CSP)
PF3D7_0324900	+	3.76E-04	erythrocyte membrane protein 1, PfEMP1
PFB0300c	+	3.59E-04	merozoite surface protein 2
PF3D7_0712300	+	3.50E-04	erythrocyte membrane protein 1, PfEMP1
PF3D7_1328200	+	3.28E-04	BRCA2 protein, putative
PF3D7_1137300	+	3.28E-04	CLPTM1 domain-containing protein, putative
PF3D7_1472100	+	3.23E-04	protein transport protein YIP1, putative
PF3D7_1215900	+	3.21E-04	serpentine receptor 10
PF3D7_0207000	+	3.19E-04	merozoite surface protein 4 (MSP4)
PF3D7_1329500	-	1.33E-03	conserved Plasmodium protein, unknown function
PF3D7_0715300	-	1.33E-03	calcium/calmodulin-dependent protein kinase, putative
PF3D7_1477500	-	1.35E-03	Plasmodium exported protein (PHISTb), unknown function
PF3D7_1402400	-	1.36E-03	zinc finger protein, putative
PF3D7_1221500	-	1.36E-03	heptatricopeptide repeat-containing protein, putative
PF3D7_1449800	-	1.37E-03	conserved Plasmodium protein, unknown function
PF3D7_0223300	-	1.39E-03	erythrocyte membrane protein 1 (PfEMP1) exon 2
PF3D7_0711700	-	1.40E-03	erythrocyte membrane protein 1, PfEMP1
PF3D7_1360400	-	1.44E-03	conserved Plasmodium protein, unknown function
PF3D7_1468300	-	1.44E-03	leucine-rich repeat protein
PF3D7_1464200	-	1.44E-03	zinc finger CCCH domain-containing protein, putative
PF3D7_1205800	-	1.45E-03	high mobility group protein B3, putative
PF3D7_1122700	-	1.46E-03	conserved Plasmodium protein, unknown function
PF3D7_0526600	-	1.46E-03	conserved Plasmodium protein, unknown function
PF3D7_0623400	-	1.49E-03	MEI2-like RNA-binding protein
PF3D7_1459000	-	1.53E-03	ATP-dependent RNA helicase DBP5
PF3D7_1454800	-	1.54E-03	conserved Plasmodium protein, unknown function
PF3D7_0223300	-	1.58E-03	erythrocyte membrane protein 1 (PfEMP1), exon 2
PF3D7_1405200	-	1.61E-03	trafficking protein particle complex subunit 1, putative
PF3D7_1002100	-	1.65E-03	EMP1-trafficking protein
PF3D7_0507700	-	1.69E-03	nuclear protein localization protein 4, putative (NPL4)
PF3D7_1240400	-	1.70E-03	erythrocyte membrane protein 1, PfEMP1
PF3D7_1410400	-	1.71E-03	rhoptry-associated protein 1 (RAP1)
PF3D7_1427600	-	1.77E-03	CorA-like Mg2 transporter protein, putative
PF3D7_0402400	-	2.44E-03	Plasmodium exported protein, unknown function

Table C: Table of top 50 identified Pf-specific antigens from the whole proteome antibody profile evaluated by ESPY at post-immunization. Here, the top 25 Pf-specific antigens associated for the protection state and the top 25 Pf-specific antigens associated for the non-protected state evaluated based on their ESPY (|d_norm|) value are listed. The effect of the evaluated Pf-specific antigen for the classification of protected versus non-protected individuals is defined by the respectively effect symbol, where '+' denotes a positive and '-' denotes a negative effect.

Plasmodb ID	effect	ESPY value (|d_norm|)	Description
PFSPZ dosis	+	5.39E-01	PFSPZ dosis
PF3D7_1123200	+	6.46E-04	leucine-rich repeat protein
PFC0210c	+	3.77E-04	circumsporozoite (CS) protein (CSP)
PF3D7_1243900	+	3.08E-04	double C2-like domain-containing protein
PF3D7_0210200	+	2.52E-04	conserved Plasmodium protein, unknown function
PF3D7_1227200	+	1.75E-04	potassium channel K1
PF3D7_1329500	+	1.68E-04	conserved Plasmodium protein, unknown function
PF3D7_0815500	+	1.52E-04	conserved Plasmodium protein, unknown function
PF3D7_0628100	+	1.44E-04	HECT-domain (ubiquitin-transferase), putative
PF3D7_1138400	+	1.08E-04	guanylyl cyclase (GCalpha)
PF3D7_1036400	+	1.04E-04	liver stage antigen 1 (LSA1)
PF3D7_1332900	+	8.53E-05	isoleucine-tRNA ligase, putative
PF3D7_1035300	+	3.87E-05	glutamate-rich protein GLURP
PF3D7_0623400	-	1.44E-03	MEI2-like RNA-binding protein
PF3D7_1107300	-	1.45E-03	polyadenylate-binding protein-interacting protein 1, putative (PAIP1)
PF3D7_1464200	-	1.45E-03	zinc finger CCCH domain-containing protein, putative
PF3D7_1402400	-	1.45E-03	Zinc finger protein, putative
PF3D7_1431900	-	1.45E-03	inner membrane complex suture component, putative
PF3D7_1411400	-	1.46E-03	cytoadherence linked asexual protein 9 (CLAG9)
PF3D7_0935800	-	1.46E-03	sphingomyelin synthase, putative
PF3D7_0625100	-	1.47E-03	60S ribosomal protein L23, putative
PF3D7_1331800	-	1.47E-03	heptatricopeptide repeat-containing protein, putative
PF3D7_1221500	-	1.48E-03	erythrocyte membrane protein 1 (PfEMP1), truncated
PF3D7_0223300	-	1.48E-03	inositol-phosphate phosphatase, putative
PF3D7_0705500	-	1.49E-03	erythrocyte membrane protein 1, PfEMP1 (VAR)
PF3D7_0711700	-	1.50E-03	conserved Plasmodium protein, unknown function
PF3D7_1328300	-	1.51E-03	conserved Plasmodium protein, unknown function
PF3D7_1449800	-	1.51E-03	leucine-rich repeat protein
PF3D7_1468300	-	1.52E-03	high mobility group protein B3, putative
PF3D7_1205800	-	1.52E-03	conserved Plasmodium protein, unknown function
PF3D7_0507700	-	1.52E-03	nuclear protein localization protein 4, putative (NPL4)
PF3D7_1454800	-	1.54E-03	conserved Plasmodium protein, unknown function
PFE1590w	-	1.56E-03	early transcribed membrane protein 5 (ETRAMP5)
PF3D7_0823900	-	1.57E-03	dicarboxylate/tricarboxylate carrier (DTC)
PF3D7_1228700	-	1.57E-03	conserved Plasmodium protein, unknown function
PF3D7_0223300	-	1.58E-03	erythrocyte membrane protein 1 (PfEMP1), exon 2
PF3D7_1329500	-	1.58E-03	conserved Plasmodium protein, unknown function
PF3D7_1422700	-	1.60E-03	conserved Plasmodium protein, unknown function
PF3D7_1450800	-	1.64E-03	conserved Plasmodium protein, unknown function
PF3D7_0526600	-	1.64E-03	conserved Plasmodium protein, unknown function
PF3D7_1405200	-	1.66E-03	trafficking protein particle complex subunit 1, putative
PF3D7_0909700	-	1.68E-03	FHA domain protein, putative
PF3D7_1427600	-	1.68E-03	CorA-like Mg2 transporter protein, putative
PF3D7_1448700	-	1.69E-03	HSP20-like chaperone, putative
PF3D7_0715300	-	1.70E-03	calcium/calmodulin-dependent protein kinase, putative
PF3D7_1240400	-	1.72E-03	erythrocyte membrane protein 1, PfEMP1
PF3D7_1410400	-	1.76E-03	rhoptry-associated protein 1 (RAP1)
PF3D7_1360400	-	1.77E-03	conserved Plasmodium protein, unknown function
PF3D7_1123400	-	1.85E-03	eukaryotic peptide chain release factor GTP-binding subunit, putative
PF3D7_1002100	-	1.91E-03	EMP1-trafficking protein
PF3D7_0402400	-	2.23E-03	Plasmodium exported protein, unknown function (GEXP18)

Table D: Table of top 50 identified Pf-specific antigens based on ESPY value from the whole proteome antibody at pre-CHMI. Here, the 12 Pf-specific antigen associated for the protection state and the top 38 Pf-specific antigens associated for the non-protected state evaluated based on their ESPY (|d_norm|) value are listed. The effect of the evaluated Pf-specific antigen for the classification of protected versus non-protected individuals is defined by the respectively effect symbol, where '+' denotes a positive and '-' denotes a negative effect. The top 12 of the positive associated and the top 38 of the negative associated Pf-specific antigens were selected based on the maximum |d_norm| value.

Plasmodb ID	effect	ESPY value (|d_norm|)	Description
PFSPZ dosis	+	6.37E-01	PFSPZ dosis
PF3D7_0712300	+	1.96E-03	erythrocyte membrane protein 1, PfEMP1
PF3D7_1101200	+	1.96E-03	rifin
PF3D7_0223100	+	1.82E-03	rifin
PF3D7_0911300	+	1.51E-03	cysteine repeat modular protein 1 (CRMP1)
PF3D7_1300100	+	1.32E-03	erythrocyte membrane protein 1, PfEMP1
PFB0300c	+	1.25E-03	merozoite surface protein 2 (MSP2)
PF3D7_1201400	+	1.10E-03	Plasmodium exported protein, unknown function
PF3D7_0601100	+	1.09E-03	exported protein family 3
PF3D7_1464500	+	9.82E-04	conserved Plasmodium membrane protein, unknown function
PF3D7_1206900	+	6.18E-04	conserved Plasmodium protein, unknown function
PF3D7_1349700	+	6.10E-04	peptidase, putative
PF3D7_1448300	+	5.67E-04	conserved Plasmodium protein, unknown function
PF3D7_0804500	+	5.03E-04	conserved Plasmodium membrane protein, unknown function
PF3D7_1136200	+	4.79E-04	asparagine-rich merozoite protein ARMA
PF3D7_0704700	+	4.39E-04	phosphopantetheine adenylyltransferase, putative
PF3D7_0725100	+	4.23E-04	conserved Plasmodium membrane protein, unknown function
PF3D7_1447800	+	4.00E-04	conserved Plasmodium protein, unknown function
PF3D7_1233400	+	3.44E-04	conserved Plasmodium membrane protein, unknown function
PF3D7_0900500	+	2.97E-04	rifin
PF3D7_1459600	+	2.54E-04	AP-4 complex accessory subunit Tepsin, putative
PF3D7_0714300	+	2.53E-04	zinc finger protein, putative
PF3D7_0614900	+	2.33E-04	conserved Plasmodium membrane protein, unknown function
PF3D7_1436100	+	1.95E-04	conserved Plasmodium membrane protein, unknown function
PF3D7_0532500	+	1.23E-04	Plasmodium exported protein, unknown function
PF3D7_1016500	+	1.06E-04	Plasmodium exported protein (PHISTc), unknown function
PF3D7_1149200	-	3.37E-03	ring-infected erythrocyte surface antigen
PF3D7_1202300	-	3.38E-03	dynein heavy chain, putative
PF3D7_1233400	-	3.40E-03	conserved Plasmodium membrane protein, unknown function
PF3D7_0711700	-	3.50E-03	erythrocyte membrane protein 1, PfEMP1 (VAR)
PF3D7_1001800	-	3.61E-03	Plasmodium exported protein (PHISTc), unknown function
PF3D7_1209300	-	3.63E-03	telomere repeat-binding zinc finger protein
PF3D7_1474600	-	3.65E-03	vacuole membrane protein 1, putative
PF3D7_0410800	-	3.65E-03	conserved Plasmodium protein, unknown function
PF3D7_1478100	-	3.67E-03	Plasmodium exported protein (hyp13), unknown function
PF3D7_1208800	-	3.73E-03	zinc finger protein, putative
PF3D7_0108300	-	3.77E-03	conserved Plasmodium protein, unknown function
PF3D7_1351300	-	3.80E-03	conserved Plasmodium membrane protein, unknown function
PF3D7_1318000	-	3.86E-03	conserved Plasmodium membrane protein, unknown function
PF3D7_0713900	-	3.99E-03	conserved Plasmodium protein, unknown function
PF3D7_1240400	-	4.00E-03	erythrocyte membrane protein 1, PfEMP1
PF3D7_0223300	-	4.20E-03	erythrocyte membrane protein 1 (PfEMP1), exon2
PF3D7_0525800	-	4.25E-03	inner membrane complex protein 1g
PF3D7_1016600	-	4.41E-03	Plasmodium exported protein, unknown function
PF3D7_1419400	-	4.44E-03	conserved Plasmodium membrane protein, unknown function
PF3D7_1459600	-	4.67E-03	conserved Plasmodium protein, unknown function
PF3D7_0718700	-	5.17E-03	conserved Plasmodium membrane protein, unknown function
PF3D7_0818500	-	5.34E-03	zinc finger protein, putative
PF3D7_0424800	-	5.51E-03	Plasmodium exported protein (PHISTb), unknown function
PF3D7_0935600	-	6.60E-03	gametocytogenesis-implicated protein (GIG)
PF3D7_0402400	-	8.74E-03	Plasmodium exported protein, unknown function (GEXP18)

Table E: Table of top 50 identified Pf-specific antigens based on ESPY value from the proteome antibody profile of pre-selected cell-surface antigens at post-immunization. Here, the top 25 Pf-specific antigens associated for the protection state and the top 25 Pf-specific antigens associated for the non-protected state evaluated based on their ESPY (|d_norm|) value are listed. The effect of the evaluated Pf-specific antigen for the classification of protected versus non-protected individuals is defined by the respectively effect symbol, where '+' denotes a positive and '-' denotes a negative effect.

Plasmodb ID	effect	ESPY value (|d_norm|)	Description
PFSPZ dosis	+	7.87E-01	PFSPZ dosis
PFC0210c	+	4.85E-04	circumsporozoite (CS) protein (CSP)
PFB0300c	+	3.60E-04	merozoite surface protein 2 (MSP2)
PF3D7_0630600	+	3.05E-04	conserved Plasmodium protein, unknown function
PF3D7_1240900	+	2.75E-04	erythrocyte membrane protein 1, PfEMP1
PF3D7_1036400	+	1.43E-04	liver stage antigen 1 (LSA1)
PF3D7_0804500	+	9.64E-05	conserved Plasmodium membrane protein, unknown function
PF3D7_1448300	+	6.51E-05	conserved Plasmodium protein, unknown function
PF3D7_1370300	-	1.59E-03	membrane associated histidine-rich protein (MAHRP1)
PF3D7_0704700	-	1.63E-03	conserved Plasmodium membrane protein, unknown function
PF3D7_1415500	-	1.63E-03	conserved Plasmodium membrane protein, unknown function
PF3D7_1436100	-	1.65E-03	conserved Plasmodium membrane protein, unknown function
PF3D7_0713900	-	1.65E-03	conserved Plasmodium protein, unknown function
PF3D7_1467000	-	1.66E-03	conserved Plasmodium membrane protein, unknown function
PF3D7_1150300	-	1.66E-03	rifin
PF3D7_0712800	-	1.67E-03	erythrocyte membrane protein 1, PfEMP1
PF3D7_1365700	-	1.69E-03	SNARE associated Golgi protein, putative
PF3D7_0600300	-	1.70E-03	rifin
PF3D7_1301600	-	1.71E-03	erythrocyte binding antigen-140
PF3D7_0400400	-	1.71E-03	erythrocyte membrane protein 1, PfEMP1
PF3D7_0731200	-	1.72E-03	Plasmodium exported protein, unknown function
PF3D7_1373000	-	1.72E-03	rifin
PF3D7_1001800	-	1.72E-03	Plasmodium exported protein (PHISTc), unknown function
PF3D7_0200600	-	1.72E-03	rifin
PF3D7_1202300	-	1.73E-03	dynein heavy chain, putative
PF3D7_0936600	-	1.78E-03	gametocyte exported protein 5
PF3D7_1202300	-	1.81E-03	dynein heavy chain, putative
PF13_0197	-	1.81E-03	merozoite surface protein 7 (MSP7)
PF3D7_1135300	-	1.84E-03	conserved Plasmodium membrane protein, unknown function
PF3D7_0912200	-	1.86E-03	conserved Plasmodium membrane protein, unknown function
PF3D7_0410800	-	1.90E-03	conserved Plasmodium protein, unknown function
PF3D7_1324300	-	1.96E-03	conserved Plasmodium membrane protein, unknown function
PF3D7_1300200	-	1.97E-03	rifin
PF3D7_0223300	-	1.97E-03	erythrocyte membrane protein 1 (PfEMP1), truncated
PF3D7_0718700	-	1.98E-03	conserved Plasmodium membrane protein, unknown function
PF3D7_1248300	-	2.00E-03	conserved Plasmodium membrane protein, unknown function
PF3D7_1347300	-	2.01E-03	conserved Plasmodium membrane protein, unknown function
PFE1590w	-	2.02E-03	early transcribed membrane protein 5 (ETRAMP5)
PF3D7_0525800	-	2.04E-03	inner membrane complex protein 1g
PF3D7_0832300	-	2.07E-03	Plasmodium exported protein (PHISTa-like), unknown function
PF3D7_1479400	-	2.07E-03	rifin
PF3D7_0730900	-	2.11E-03	Plasmodium exported protein, unknown function
PF3D7_1149200	-	2.12E-03	ring-infected erythrocyte surface antigen
PF3D7_0712000	-	2.14E-03	erythrocyte membrane protein 1, PfEMP1
PF3D7_0935600	-	2.15E-03	gametocytogenesis-implicated protein (GIG)
PF3D7_0223300	-	2.16E-03	**erythrocyte membrane protein 1 (PfEMP1), exon 2**
PF3D7_0818500	-	2.17E-03	zinc finger protein, putative
PF3D7_1229000	-	2.23E-03	conserved Plasmodium membrane protein, unknown function
PF3D7_0424800	-	2.28E-03	Plasmodium exported protein (PHISTb), unknown function
PF3D7_1240400	-	2.31E-03	erythrocyte membrane protein 1, PfEMP1
PF3D7_0402400	-	3.23E-03	Plasmodium exported protein, unknown function (GEXP18)

Table F: Table of top 50 identified Pf-specific antigens based on ESPY value from the proteome antibody profile of pre-selected cell-surface antigens at pre-CHMI. Here, seven Pf-specific antigens associated for the protection state and the top 43 Pf-specific antigens associated for the non-protected state evaluated based on their ESPY (|d_norm|) value are listed. The effect of the evaluated Pf-specific antigen for the classification of protected versus non-protected individuals is defined by the respectively effect symbol, where '+' denotes a positive and '-' denotes a negative effect.
